# Genomics Reveals Complex Population History and Unexpected Diversity of Eurasian Otters (*Lutra lutra*) in Britain Relative to Genetic Methods

**DOI:** 10.1093/molbev/msad207

**Published:** 2023-09-15

**Authors:** Sarah J du Plessis, Mark Blaxter, Klaus-Peter Koepfli, Elizabeth A Chadwick, Frank Hailer

**Affiliations:** School of Biosciences, Cardiff University, Cardiff, UK; Tree of Life, Wellcome Sanger Institute, Cambridge, UK; Smithsonian-Mason School of Conservation, George Mason University, Front Royal, VA, USA; Centre for Species Survival, Smithsonian's National Zoo and Conservation Biology Institute, Washington, DC, USA; School of Biosciences, Cardiff University, Cardiff, UK; School of Biosciences, Cardiff University, Cardiff, UK

**Keywords:** population genomics, bottleneck, demographic history, reintroductions, genetic tools

## Abstract

Conservation genetic analyses of many endangered species have been based on genotyping of microsatellite loci and sequencing of short fragments of mtDNA. The increase in power and resolution afforded by whole genome approaches may challenge conclusions made on limited numbers of loci and maternally inherited haploid markers. Here, we provide a matched comparison of whole genome sequencing versus microsatellite and control region (CR) genotyping for Eurasian otters (*Lutra lutra*). Previous work identified four genetically differentiated “stronghold” populations of otter in Britain, derived from regional populations that survived the population crash of the 1950s–1980s. Using whole genome resequencing data from 45 samples from across the British stronghold populations, we confirmed some aspects of population structure derived from previous marker-driven studies. Importantly, we showed that genomic signals of the population crash bottlenecks matched evidence from otter population surveys. Unexpectedly, two strongly divergent mitochondrial lineages were identified that were undetectable using CR fragments, and otters in the east of England were genetically distinct and surprisingly variable. We hypothesize that this previously unsuspected variability may derive from past releases of Eurasian otters from other, non-British source populations in England around the time of the population bottleneck. Our work highlights that even reasonably well-studied species may harbor genetic surprises, if studied using modern high-throughput sequencing methods.

## Introduction

### Genetic and Genomic Methods

As molecular ecology expands to include whole genome sequencing (WGS), the congruence between genetic and genomic methods has been called into question (McCartney-Melstad et al. 2018; Zimmerman et al. 2020). Rather than discrete categories, genetic and genomic methods form a spectrum across marker type, number of loci, and technologies used to generate data. Generally, methods using fewer than a thousand loci are considered genetic and therefore include microsatellites and mitochondrial fragments that are usually generated using Sanger sequencing–based methods (Hohenlohe et al. 2021). Methods using thousands or more loci are considered genomic, therefore including WGS and reduced representation methods such as restriction site-associated DNA sequencing (RADseq), which are produced using high-throughput sequencing technologies (Supple and Shapiro 2018; Hohenlohe et al. 2021). There is a trade-off in data acquisition between the number of individuals that can be assayed and the number of loci at which variation in each individual can be measured. WGS is becoming increasingly affordable but is still effectively limited to relatively small sample sizes. To maximize the potential benefit from the array of methods on the genetics–genomics continuum, an understanding of when these results are likely to be congruent, or differ, will enable the most cost-effective marker selection for the specific research and conservation project (Gallego-García et al. 2021).

Ascertainment bias, the nonrandom analysis of loci resulting in parameter estimate biases (Nielsen 2000), has the potential to affect both genetic and genomic methods. For instance, microsatellite selection based on a single population will be biased toward detection of variation present in that population and away from detection of variation in a distinct, highly differentiated population (Malomane et al. 2018). This results in systematic deviations, such as the underrepresentation of rare alleles, which can underestimate signals of a population expansion (Nielsen 2000). Genomic methods such as RADseq and WGS are less susceptible to ascertainment bias, due to the largely random choice of loci. Furthermore, if few loci show strong bias, these will have a larger effect in smaller, genetic data sets than in larger, genomic data sets.

Due to the varying evolutionary rates of different markers, more loci or greater length of sequence are required to obtain comparable levels of resolution and hence statistical power. For example, microsatellites are chosen to be multiallelic with an unusually high and variable mutation rate (from 10^−3^ to 10^−4^ mutations per locus per generation), whereas single nucleotide polymorphisms (SNPs) are primarily biallelic and have a lower mutation rate of 10^−8^–10^−9^ per nucleotide per generation (Ellegren 2000; Väli et al. 2008). Microsatellites provide an effective method of identifying significant differences in variability even with low numbers of loci, and SNPs are often assessed in the thousands to millions in genomic data sets. Microsatellite loci are assumed to be largely neutral and thus useful for assessing genetic diversity and structure, whereas approaches such as WGS provide the opportunity to investigate the evolutionary mechanisms behind the measures of genetic variation, such as inbreeding depression, genetic basis of adaptation, and functional variation (Supple and Shapiro 2018; Hohenlohe et al. 2021).

It is important to evaluate characteristics of the study population when considering variation in results between methods. The variation found within species with large effective population sizes (e.g., *N*_e_ > 1,000) is unlikely to be accurately quantified through analysis of a few loci. This issue is less critical in analyses of divergent, small, and inbred populations (Gallego-García et al. 2021). Furthermore, the number of samples taken to represent a population will constrain the power of analyses. For example, the potential to detect fine-scale population structuring is limited with few samples and the generally small sample sizes typical of genomic approaches may not yield the same insights as those made with much larger genetic sample size (Gallego-García et al. 2021). A clear benefit of genomic approaches is the ability to standardize results across research groups and therefore directly compare results between broad populations and species. For example, using a standard reference genome and short-read WGS data analyzed with the same bioinformatic pipeline facilitates direct comparison of data, which is rarely possible using microsatellite markers.

### Case Study: Eurasian Otter (*Lutra lutra*)

Eurasian otter (*L. lutra*) populations in Britain suffered a substantial population crash between 1950 and 1980 due to environmental chemical contaminants, such as persistent organic pollutants (Chanin and Jefferies 1978). This pattern was broadly observed across Europe, resulting in the species being classified as “near threatened” on the International Union for Conservation of Nature (IUCN) Red List (Roos et al. 2015). Although there are only limited direct estimates of otter population sizes throughout this time period, otter hunting records provide a proxy for population size. These indicate drastic population declines across southwest England and less drastic declines in northern England and Scotland from 1957 (Chanin and Jefferies 1978), highlighting a potential variation in the impact of chemical contaminants across the landscape. Restrictions and bans on the use of these chemicals in the 1970s lead to population recovery (Walker and Newton 1998; Mason and Macdonald 2004), as tracked by national surveys beginning in 1977 across Britain. The percentage of sites visited showing signs of otters increased from 6% to 59% in England, from 20% to 89% in Wales, and from 61% to 92% in Scotland, when comparing surveys from 1977 to 2009 for England and Wales and 1977 to 2003 for Scotland. The species has broadly been acclaimed as a conservation success story (Macdonald 1983; Strachan et al. 1990; Crawford and Scholey 2010; Kean and Chadwick 2021). However, the most recent National Survey of Wales identified signals of a recent, substantial population decline, highlighting the importance of continual monitoring and the value of otters as an indicator species for the ecological health of aquatic systems of Britain (Kean and Chadwick 2021).

Since the 1950–1980 population crash, extensive genetic assessments have been conducted on *L. lutra* in Britain, using both microsatellites (Dallas and Piertney 1998; Dallas et al. 1999, 2002; Hobbs et al. 2006, 2011; Stanton et al. 2014; Thomas et al. 2022) and mitochondrial fragments (Stanton et al. 2009). These data have been incorporated into broader European assessments using both marker types (Cassens et al. 2000; Randi et al. 2003; Ferrando et al. 2004; Mucci et al. 2010). These studies illustrate the consequences of the population crash by revealing distinct genetic population strongholds. They were also critical in developing and sharing optimized microsatellite methods (Hobbs et al. 2011). A culmination of this work was a microsatellite data set spanning 21 years of the population recovery in Britain, which identified a time lag in the genetic connectivity between populations (Thomas et al. 2022).

**Fig. 1.**
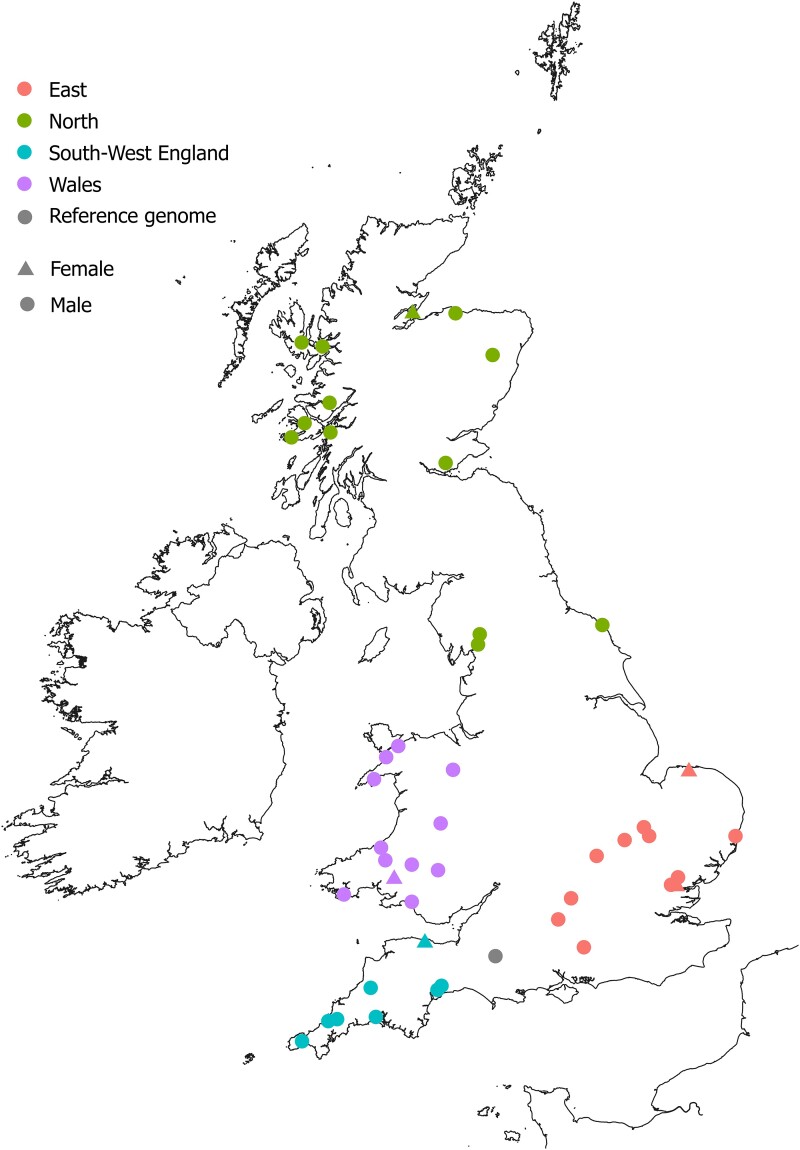
Geographic origins of Eurasian otter samples analyzed in this study and the reference genome.

Over 4,000 otters from across Britain, primarily victims of road traffic accidents, have been collected and preserved by the Cardiff University Otter Project since 1994, most of which are suitable for genomic analysis. A male *L. lutra* from Somerset, Southwest England, was sequenced and assembled to chromosomal completeness by the 25 genomes for 25 years project at the Wellcome Sanger Institute (Mead et al. 2020), and this high-quality reference genome is a strong backbone on which data can be mapped and contextualized. The Eurasian otter in Britain therefore presents an ideal opportunity to directly compare inferences derived from parallel data sets generated from genetic and genomic methods. As the first population genomic study of the species, we addressed two objectives. First, we used both WGS and an established microsatellite panel to directly compare population metrics derived from genomic and genetic methods for the same sample set of Eurasian otters in Britain (fig. 1). Secondly, we used the genomic data to assess inbreeding and historic effective population size estimates for the first time. We predicted that:

1. Detected genetic variation and population structuring will differ between genetic and genomic methods.
2. The recent, anthropogenic population bottleneck (which was not previously identified using genetic methods) will be identifiable from genomic signatures, such as runs of homozygosity (ROH), linkage disequilibrium (LD), and changes in effective population size over time.

## Results

### Population Structure Using Genetic and Genomic Data

**Fig. 2.**
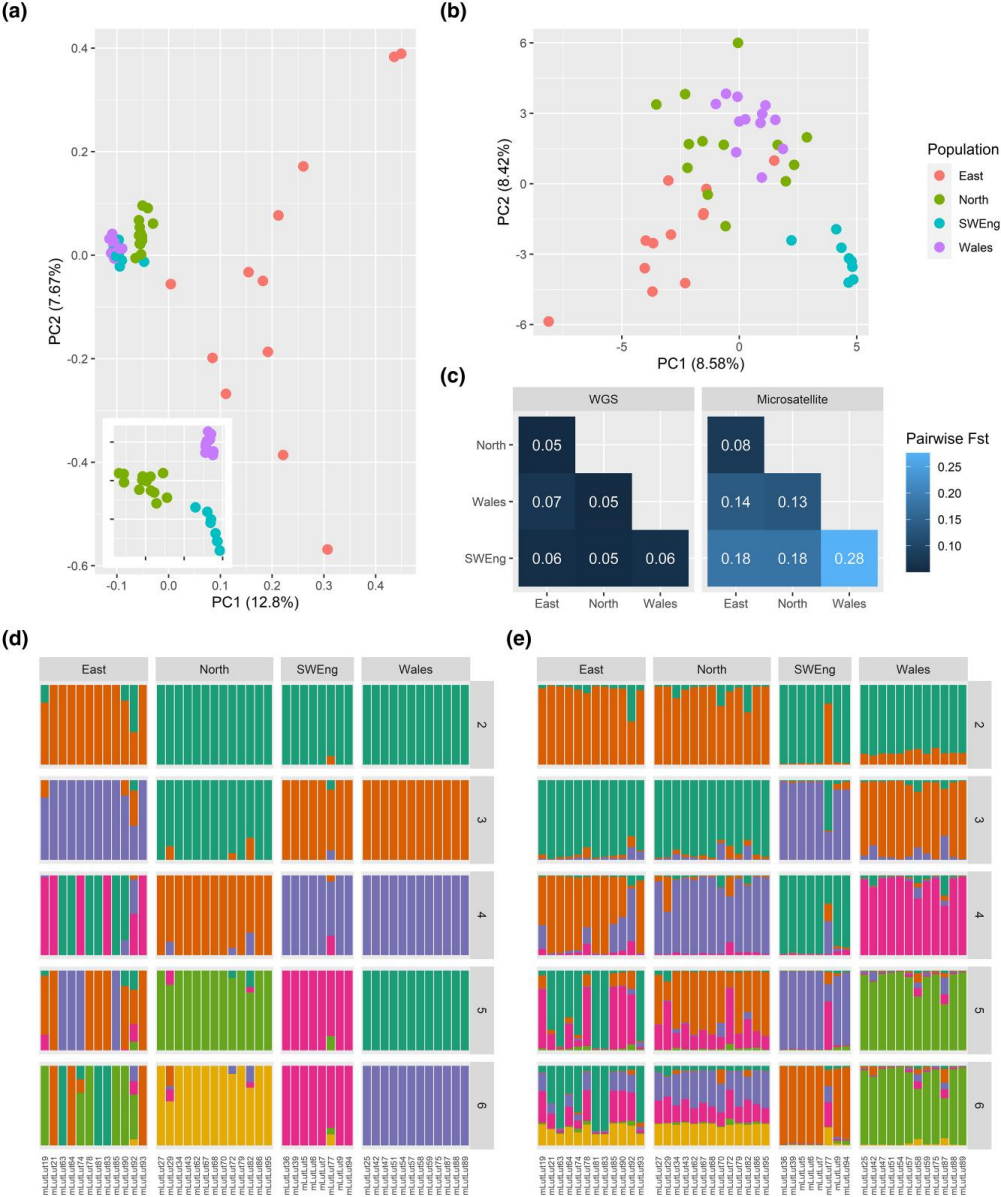
Comparison of estimates of population structuring of Eurasian otters in Britain, based on WGS versus microsatellite data. PCAs of (*a*) whole genome SNPs and (*b*) microsatellites for the whole data set and (in the inset) for the 33 non-Eastern samples and (*c*) pairwise *F*_ST_ values for whole genome SNPs and microsatellites. (*d*) ADMIXTURE results for whole genome SNPs and (*e*) STRUCTURE results for microsatellites.

Principal component analysis (PCA) of SNPs from the whole genome resequencing data (fig. 2*a*) was dominated on both PC1 (12.8%) and PC2 (7.7%) by variation among samples within the East population. Removing samples from the East population and repeating the PCA of SNP data revealed geographic clustering of samples from the remaining three populations (fig. 2*a* inset). In the PCA of the microsatellite data from the same specimens (fig. 2*b*), PC1 accounted for 8.6% of variation in the data and PC2 8.4%, without clear separation of samples by population, except for the Southwest England population. Pairwise *F*_ST_ values among the four populations were significantly higher in the microsatellite than the whole genome data set, with microsatellite-derived values ranging from 0.08 to 0.28 and SNP-derived values from 0.05 to 0.07 and a mean difference in *F*_ST_ between methods of 0.11 (paired *t*-test, t_5_ = 3.97, *P* = 0.01; fig. 2*c*).

We explored likely population substructuring in whole genome SNP and microsatellite data. The cross-validation (CV) error of ADMIXTURE analyses on the SNP data indicated the most likely value of *K* to be 1; however, the delta *K* of STRUCTURE analyses of the microsatellite data indicated the most likely value of *K* to be 3 (supplementary material S2.6, Supplementary Material online). Due to an increase in discordance among replicate STRUCTURE analyses and varying individual cluster assignments of the ADMIXTURE analyses at higher *K* values, results up to *K* = 6 are presented for both methods in fig. 2*d* and *e*, whereas the results from the full range of *K* values are shown in supplementary material S2.6, Supplementary Material online. The STRUCTURE plot of microsatellite data indicated more admixture among populations than identified by ADMIXTURE on whole genome SNP data. Samples clustered broadly by geographic population in the STRUCTURE plot at *K* = 4, whereas in the ADMIXTURE analysis at *K* = 4, there was substructuring in the East, and Southwest England clustered with Wales. Southwest England and Wales were separated at higher *K* values. However, substructuring in the East was inconsistent from *K* = 4 to *K* = 6, with varying population assignments of the same individuals at increasing *K* values, consistent with the absence of pronounced substructuring within the East in the PCA (fig. 2*a*). No pairwise relatedness of third degree or closer was identified among the samples (supplementary material S2.7, Supplementary Material online).

To probe the complex population assignments of individuals within the East population at increasing levels of *K*, fineSTRUCTURE analyses were run on the SNP data. The fineSTRUCTURE coancestry matrix (supplementary material S2.6, Supplementary Material online) showed clear population structuring between the East and the remaining populations, with less distinct population structuring between the Wales and North and Southwest England populations. The matrix also suggested lower levels of shared coancestry between the East and other populations, compared to within and between the non-Eastern populations. In contrast to the ADMIXTURE results, fineSTRUCTURE clustered mLutLut92 (East) with samples from the North.

### Genomic Diversity within Britain

**Fig. 3.**
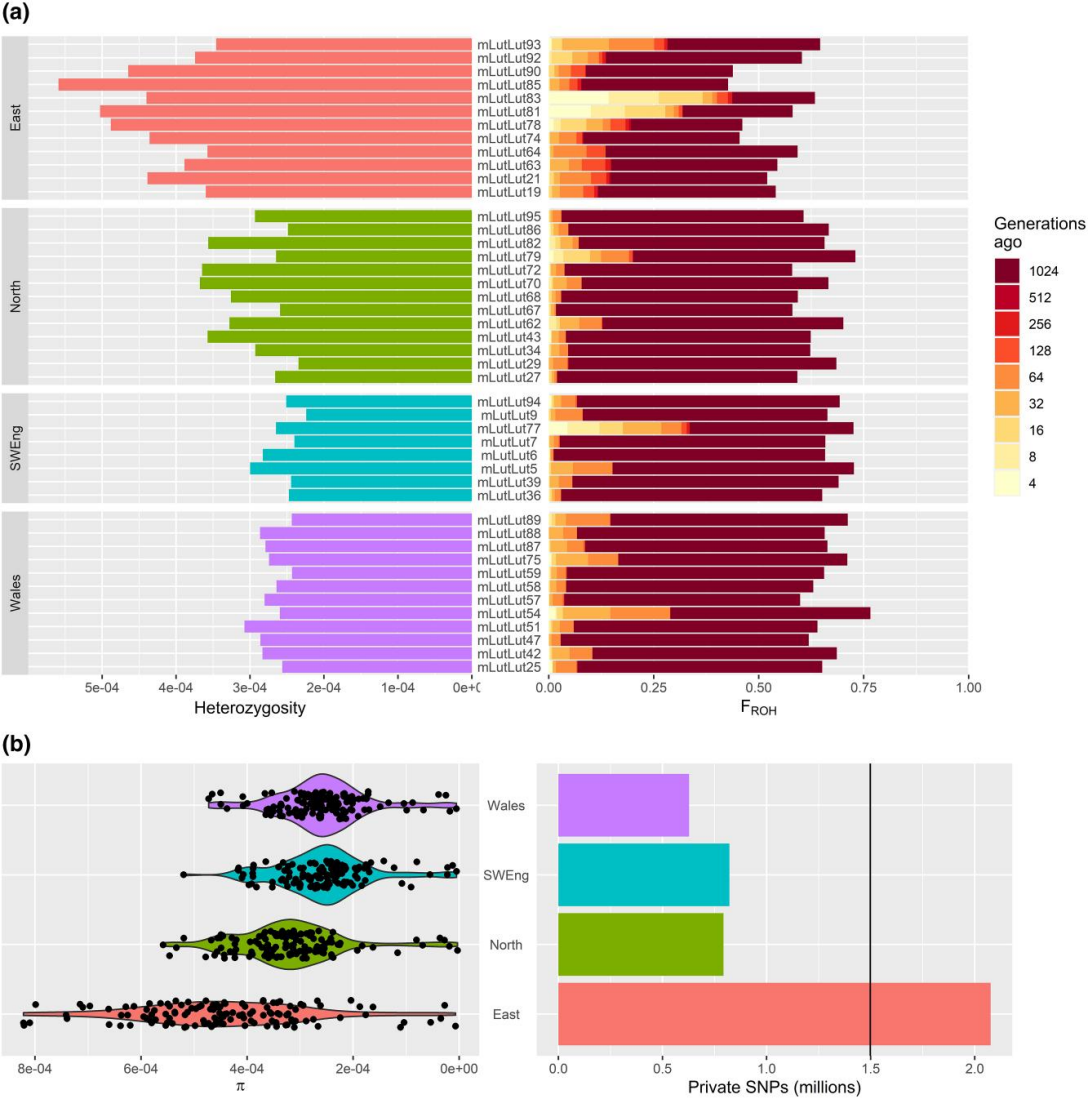
Genomic diversity in British Eurasian otters based on SNP markers. Individual-level (*a*) genome-wide heterozygosity and proportion of the genome in runs of homozygosity (*F*_ROH_) as calculated by RzooROH, color-coded by number of generations ago inbreeding is estimated to have occurred (from older runs/shorter ROH to recent/longer ROH). Population-level measures (*b*): nucleotide diversity (π) calculated in 20 Mb sliding windows and counts of SNPs private to each population (bars), relative to the number of SNPs common to all populations (black line at 1,498,476 SNPs).

Based on whole genome SNP data, heterozygosity was higher in the East population than in other populations (mean difference of 0.00013–0.00017 higher in the East; *F*_3,41_ = 33.43, *P* < 0.0001; fig. 3*a*). The realized inbreeding coefficient (*F*_ROH_) based on the proportion of genomes in ROH ranged from 0.43 to 0.77 and was significantly lower in the East, with no significant differences among the remaining populations (*F*_3,41_ = 16.21, *P* = 0.0004; fig. 3*a*). The majority of ROH fell into the shortest, and therefore oldest class, indicating that extensive inbreeding occurred at 1,024 or more generations ago (an estimated 4,096 years). Signals of longer ROH (from 4 and 8 generations, or 16 and 32 years ago) were visible in a few individuals in the East and scarcer in the remaining populations.

Nucleotide diversity (π) was significantly higher across genomic windows in the East and significantly different among all population pairs except Southwest England and Wales (*F*_3,504_ = 76.61, *P* < 0.0001; fig. 3*b*). Private SNPs showed the same trend, with more SNPs identified as private to the East (2,076,325) than were private to any other population or common across all populations (1,498,476; fig. 3*b*; full private SNPs results in supplementary material S2.8, Supplementary Material online).

LD decay over distance (up to 100 kb) showed varying trends among populations (supplementary material S2.8, Supplementary Material online). Southwest England consistently showed the highest overall levels of LD and the slowest decline with increasing chromosomal distance; the North showed consistently lowest overall levels of LD, with the quickest decline over increasing chromosomal distance. Wales and East showed similar patterns of LD decay, which varied among chromosomes (supplementary material S2.8, Supplementary Material online).

### Genetic Diversity within Britain

Genetic diversity statistics based on microsatellites were within the range of values reported in previous microsatellite studies (Thomas et al. 2022) despite the lower sample size in the present study. Observed heterozygosity based on microsatellites was highest in the North and East (0.64 and 0.63), followed by Wales (0.53), and the lowest observed heterozygosity was identified in Southwest England (0.48). Since these statistics are not comparable across methods, we report the population rankings and full results in supplementary material S2.5, Supplementary Material online. When populations are ranked from highest to lowest heterozygosity, rankings varied between data sets: the whole genome SNP results rank East > North > Wales > Southwest England, whereas microsatellites rank North > East > Wales > Southwest England. Private alleles were identified in 12 of the 15 microsatellite loci, with 10 alleles across all loci private to the East, 11 alleles private to the North, and only 1 allele private to Southwest England. No alleles were found only in Wales (see supplementary material S2.5, Supplementary Material online). Rankings for *F*_IS_/*F*_ROH_ showed a more notable difference, with whole genome SNP results ranking Southwest England > Wales > North > East and microsatellites ranking East > North > Southwest England > Wales.

### Historic *N*_e_

**Fig. 4.**
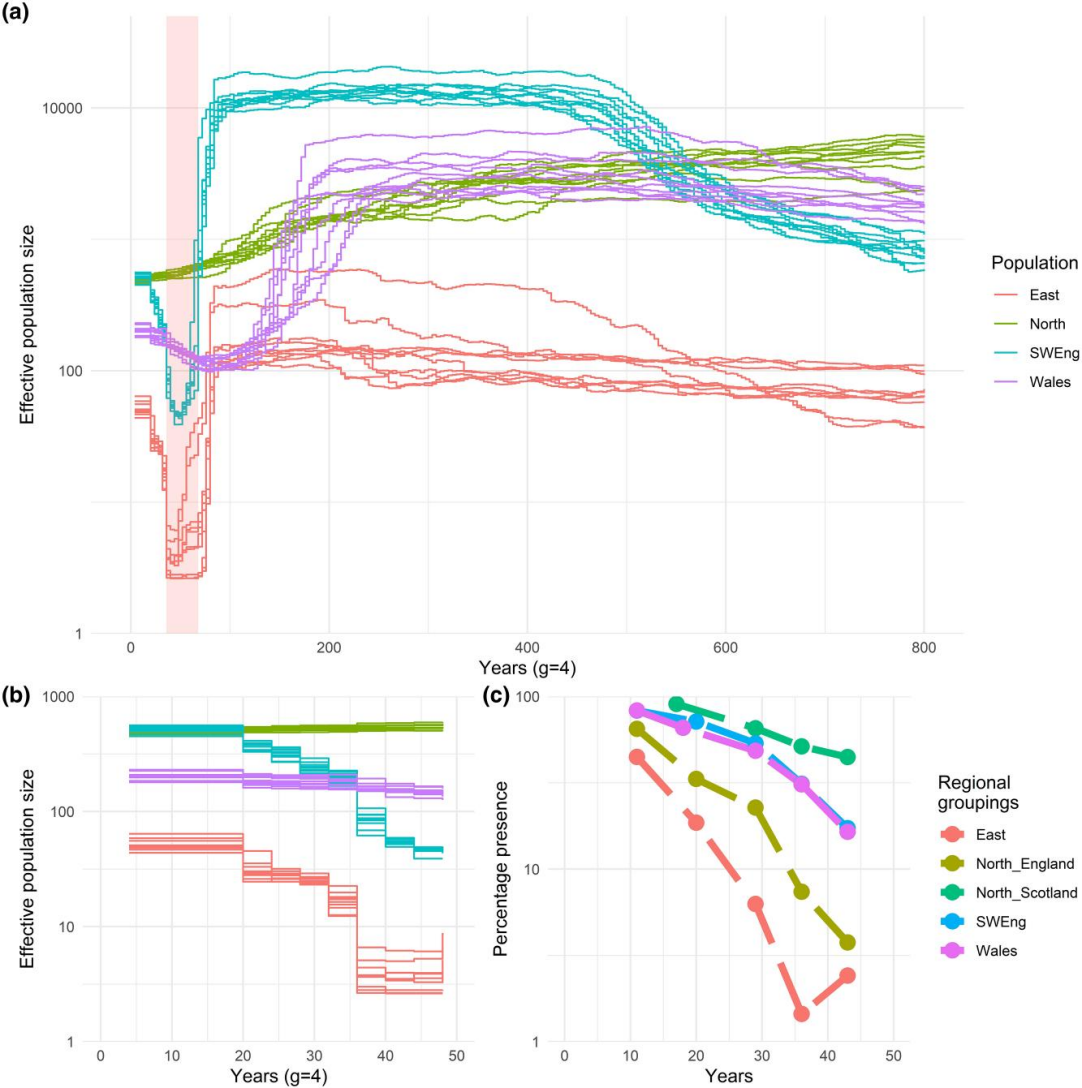
Estimated effective population size through time by population of Eurasian otter in Britain based on SNP data and comparable survey data. (*a*) GONE analyses were conducted on a population-level scale, with ten bootstrap repeats for each population, assuming a generation time of 4 years, conducted on samples sizes of *n* = 8 for Southwest England, *n* = 12 for Wales and East, and *n* = 13 for North, and the expected timing of the bottleneck highlighted (1950–1970). (*b*) GONE analyses for the same period of time for which surveys were conducted (0–50 years before samples were collected in 2020). (*c*) Survey data as percentage of sites surveyed which showed positive signs of otters, grouped by regions reflecting stronghold populations, with the exception of the North, which was split into regions from England and Scotland based on divergent census size trends. For surveys which spanned more than 1 year, the results are plotted at the earliest year. All plots are presented on a log scale of *N*_e_.

Historic effective population size (*N*_e_) was estimated using GONE for the recent past (up to 800 years ago) and pairwise sequentially Markovian coalescent (PSMC) for the ancient past (10,000 to 1,000,000 years ago) (fig. 4*a*; supplementary material S2.9, Supplementary Material online). The demographic population bottleneck was expected to have occurred between the 1950s and 1970s, and this expectation corresponded very accurately to the declines and recoveries of *N*_e_ estimated by GONE. Both the East and Southwest England populations showed substantial bottlenecks and recoveries between the 1950s and 1970s, but the *N*_e_ of the Southwest was consistently higher than that of the East, which declined to 3.7 from 1972 to 1984 (averaged across bootstrap *N*_e_ estimates for 9–12 generations ago). The decline in *N*_e_ started earlier in Wales, during the 1800s, and showed an increase in the past 50 years. The North showed a gradual, continual decline through the past 800 years. For the most recent 50 generations, we compared *N*_e_ estimates using GONE (fig. 4*b*) to the survey data recording the number of sites showing positive signs of otters by region (fig. 4*c*). Visually, the trends matched reasonably well, with the differences in trends between regions and the extremely low proportion presence in the East coinciding with the low *N*_e_ estimates. At deeper timescales, PSMC analyses of 10,000 to 1,000,000 years ago showed a decline in *N*_e_ across all populations, albeit with some local variation.

### Mitochondrial Genomes versus Control Region

**Fig. 5.**
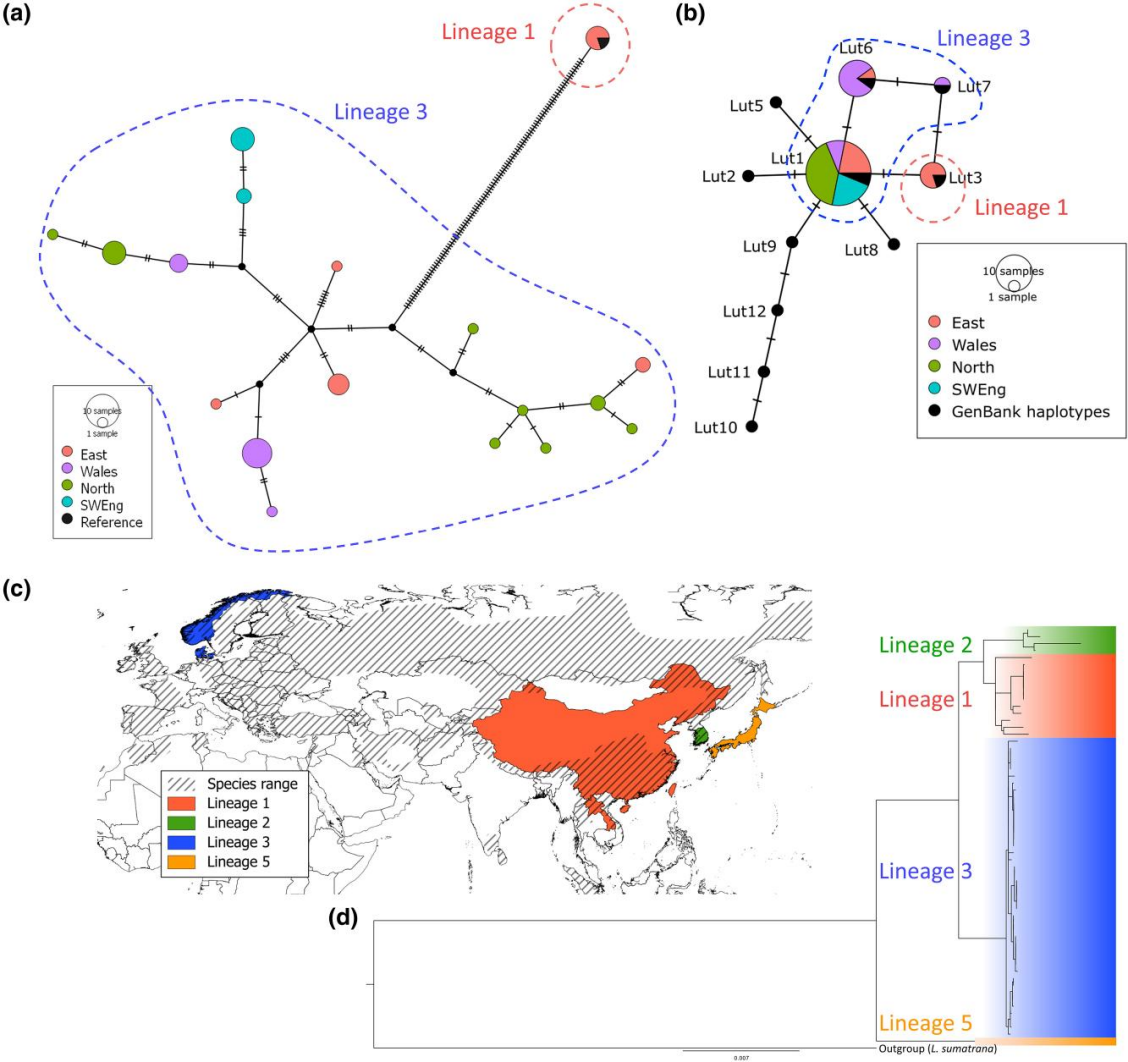
TCS networks, phylogeny, and locations of lineages of mitochondrial genome variation in Eurasian otters. (*a*) TCS network of 44 British samples and the reference mitochondrial genome (LR822067.1), based on 16,365 bp mitochondrial sequence (with the repeat region removed). (*b*) TCS network of the same 44 British samples and known CR haplotypes from GenBank (in black). Lut4 and mLutLut29 are collapsed into Lut1, as they only differ by a single base indel. (*c*) Countries shaded to indicate where each whole mitochondrial genome lineage was identified, and hatching indicates known, global Eurasian otter range. Japan included both lineages 1 and 5 and therefore is shaded orange for the unique lineage 5. No colored shading is shown for Russia and Britain in this diagram, due to multiple lineages identified (see supplementary material S2.11, Supplementary Material online). (*d*) Phylogenetic tree of 16,392 bp whole mitochondrial sequence (with the repeat region removed) generated in this study (*n* = 44), assembled from SRA (*n* = 3), and downloaded from GenBank (*n* = 13), rooted with a hairy-nosed otter (*L. sumatrana*), totaling 61 sequences. Posterior probabilities of branches between lineages are all 1. Tree shading indicates lineage 1 (red), 2 (green), 3 (blue), and 5/*L. l. nippon* (orange). Full phylogeny and posterior probabilities are given in supplementary material S2.11, Supplementary Material online.

Of the 45 British samples, whole mitochondrial genomes for 44 were assembled, of which 41 were assembled using NOVOPlasty and 3 were assembled using MITObim (table 1). From these 44 sequences, 18 unique haplotypes were identified across 153 segregating sites (summary statistics and haplotypes given in supplementary materials S1 and S2.11, Supplementary Material online, respectively). The TCS network identified 2 distinct lineages, equivalent to lineages 1 and 3 in the lineage classification of Waku et al. (2016), separated by a branch representing 105 mutations between groups (fig. 5*a*). Due to the presence of both divergent lineages in the East, the number of segregating sites and nucleotide diversity were higher in the East than other British populations; however, haplotype diversity was highest in the North (supplementary material S2.11, Supplementary Material online).

**Table 1.** Eurasian Otter Sample Accessions, Locations, and Publication Source of Publicly Available SRA and Whole Mitochondrial Genome Sequences Incorporated into Analyses.

Sample Accession	Location	Publication	Lineage
SRA reads downloaded and assembled:			
SRR19383068	Narvik, Norway	de Ferran et al. (2022)	^a^Lineage 3
SRR19383067	Tyumen Oblast, Russia	de Ferran et al. (2022)	^a^Lineage 3
SRR11679564	Denmark	Margaryan et al. (2021)	^a^Lineage 3
Whole mitochondrial genome sequence downloaded:			
LC049377	China	Waku et al. (2016)	Lineage 1
LC049378	China	Waku et al. (2016)	Lineage 1
LC049952	Sichuan, China	Waku et al. (2016)	Lineage 1
LC049953	Unknown	Waku et al. (2016)	Lineage 1
LC049954	Sakhalin, Russia	Waku et al. (2016)	Lineage 2
LC049955	Kanagawa, Japan	Waku et al. (2016)	Lineage 1
LC050126	Kochi, Japan	Waku et al. (2016)	*L. l. nippon*/^a^lineage 5
LC094961	Laos	Waku et al. unpublished	^a^Lineage 1
LR822067/NC_062277	Southwest England	Mead et al. (2020)	^a^Lineage 1
MW316682	Kinmen, Taiwan	Jang-Liaw et al. unpublished	^a^Lineage 1
EF672696	Korea	Ki et al. (2010)	^a^Lineage 2
FJ236015/NC_011358	Korea	Jang et al. (2009)	Lineage 2
MW573979	Daejeon, South Korea	Kim and Jo (2021)	^a^Lineage 2
KY117556	—	Mohd Salleh et al. (2017)	*L. sumatrana*

Note.—Lineage names and sample allocation as defined by Waku et al. (2016).

^a^Assigned in the present study.

All five control region (CR) haplotypes which have previously been identified in Britain were found in our data set. Haplotypes Lut1, Lut3, Lut6 (Stanton et al. 2009), Lut4 (Cassens et al. 2000), and Lut7 (Pountney 2008) were found in 29, 4, 9, 1, and 1 samples, respectively. The difference between Lut1 and Lut4 is a single base indel at nucleotide position 96, and therefore, these haplotypes were collapsed in the haplotype network (fig. 5*b*). Geographic distribution of these haplotypes was broadly consistent with previous findings. For example, Lut1 was found across all populations, whereas Lut3 was only identified in the East. Interestingly, however, Lut6, previously only found in Western Britain, was identified in eight samples from Wales and one sample from the East in our data set. Only the four samples belonging to lineage 1 corresponded to CR haplotype Lut3, separated from the remaining haplotypes by a single mutation.

We also assembled mitochondrial genomes from three previously generated short-read data for European *L. lutra* available in the short-read archive. These read sets were successfully assembled using NOVOPlasty and aligned with European and Asian *L. lutra* mitochondrial genome sequences from GenBank (*n* = 13) and the British samples generated in this work, yielding a total of 60 *L. lutra* sequences. We added a single hairy-nosed otter (*Lutra sumatrana*) as outgroup. From the 60 *L. lutra* mitochondrial genomes, 34 unique haplotypes were identified across 772 segregating sites. Phylogenetic analysis showed separation of samples into four main lineages, all with posterior probabilities of 1.00 (fig. 5*d*; sample lineages in supplementary material S2.11, Supplementary Material online). Three lineages were named by Waku et al. (2016) as *L. l. nippon* (here referred to as lineage 5), lineage 1, and lineage 2, and one new lineage was identified in this study (lineage 3). Geographic locations of these lineages are provided in fig. 5*c*, with the exception of Britain and Russia for which multiple lineages were identified. The British samples sequenced in this study identified two divergent lineages: lineage 1 and lineage 3. Sequences assigned to lineage 3 include 40 samples from Britain (across all populations) and the SRA-derived sequences from Denmark, Russia, and Norway. Lineage 1 included four samples from the East of Britain, alongside GenBank samples from China, Laos, and Japan, and the reference genome sequence, from a British otter (from Somerset, closest geographically to our Southwest England region). Although the rooted phylogenetic tree indicated that lineage 5/*L. l. nippon* split first, followed by lineage 3 and then lineages 1 and 2, variation in sequences within lineage 3 appeared to be a recent diversification relative to the older branching within lineages 1 and 2. These results were reflected in the higher number of segregating sites and nucleotide diversity observed within lineages 1 and 2, relative to lineage 3.

## Discussion

### Genetic versus Genomic Methods

Our study provides a direct comparison of genetic and genomic methods in assessment of genetic diversity and population structure in a threatened wild carnivore, the Eurasian otter. We applied genomic methods to investigate the occurrence and effects of a recent, anthropogenically driven population decline and recovery of Eurasian otters in Britain. Our concurrent analysis of microsatellite and SNPs from the same sample set highlights the complexities inherent in interpreting results of these approaches.

Broadly, our microsatellite and SNP data sets are not concordant, with substantial differences in the order of population differentiation and population groupings. For example, at *K* = 3, the SNP data set grouped Southwest England and Wales, whereas the microsatellite data grouped East and North England. Across all samples, the proportion of admixture identified is higher in the microsatellite data set than the SNP data. However, for the purpose of population assignment based on a suitable *K* value, there is also some concordance across data sets, with the exception of some samples between the East and North in the microsatellite data. To summarize, similarities in the ADMIXTURE/STRUCTURE comparison broke down at higher values of *K* and in more complex scenarios such as the substructuring within the East. Similarly, SNPs showed more population structuring in PCA, and microsatellites showed significantly higher pairwise *F*_ST_ estimates. These results are not unexpected, as both similarities (Zimmerman et al. 2020) and differences (Lah et al. 2016) such as these have been identified in past studies of comparable genetic and genomic data sets.

Both the number of loci assessed and ascertainment bias are likely to be contributing to the differences between the genetic and genomic approaches. Specifically, the higher resolution of data captured by almost 9 million biallelic SNPs, compared with 15 multiallelic microsatellites, is likely to be critical, as in several prior studies (Lah et al. 2016; Natesh et al. 2017; Lavretsky et al. 2019; Gallego-García et al. 2021). Although the microsatellites identified the broad patterns of population structuring accurately, they do not hold the same power to detect more fine-grained population distinction (higher values of *K*) and more complex scenarios, when compared with the genomic results. Since the complexity of any given study system is typically unknown, it is important to recognize that conclusions based on genetic methods may change following the application of genomic data. In particular, the measures which showed the most significant differences between methods (e.g., pairwise *F*_ST_) are likely to be inflated due to ascertainment bias of the microsatellite loci. Specifically, these microsatellite loci were identified and selected based on the variation they identified in Scotland and Wales (both sampled in this study), and therefore, they are likely to represent only a subset of the variation present across populations in Britain (Dallas and Piertney 1998; Dallas et al. 1999; Hobbs et al. 2006). Although ascertainment bias is less commonly identified as a source of variation between genetic and genomic comparisons, Fischer et al. (2017) showed significantly larger estimates of pairwise *F*_ST_ using microsatellites than SNPs, which they attributed to the ability of high-coverage WGS in identifying rare variants, which reduce overall *F*_ST_. Similarly, Cairns et al. (2023) identified higher proportions of admixture among dogs and dingoes (*Canis* spp.) using microsatellites relative to SNPs, where the microsatellites had been selected based on allele frequencies identified in dogs. Taken together, these findings highlight potential impacts of microsatellite ascertainment bias, leading to inflated admixture proportions (Cairns et al. 2023).

Our utilized microsatellite panel does not contain any loci pairs with significant LD signals, and all loci are at least 1.34 Mbp apart in the genome. Consequently, linkage appears unlikely to have had a large effect on our microsatellite results. Linkage signals are higher for our analyzed SNPs, due to their larger numbers (9 million) and greater proximity in the genome. Therefore, although linkage occurs in both data sets, it is likely to be affecting the genomic results more significantly than the genetic results in this study. At this point, it becomes difficult to disentangle the effects of number of loci, ascertainment bias, and linkage, as using too few loci or using nonrandomly selected loci could both underrepresent rare alleles and therefore lead to inflated *F*_ST_ estimates, among other effects, further supporting the crucial role of genomic data.

We are not suggesting microsatellite markers are redundant, and we emphasize the important role they continue to play as an affordable marker system for cluster assignment and other scenarios. Equally, long-term genetic data sets, such as Thomas et al. (2022), highlight the importance of the continuity of markers to assess temporal trends in genetic variation, for example, during population recovery and expansion. However, our analyses suggest that due to numbers of loci and ascertainment bias, the absolute values of metrics calculated based on microsatellites should be interpreted with caution, with a focus on relative values (such as a decrease in *F*_ST_ over time) likely being much more informative. Importantly, the scale of the effect of ascertainment bias varies depending on the specific loci and samples, and therefore, comparisons between studies based on different loci, populations, samples, or species would be inappropriate.

Using whole mitochondrial genomes, we were able to identify two divergent lineages within Britain, which were not previously identified based on CR studies (Stanton et al. 2009), and were not possible to identify using CR fragments for our samples. Based on these results, we strongly recommend that analyses using short mitochondrial fragments should be upgraded to whole mitochondrial genome analyses where possible, to avoid misleading inferences.

### Demographic Analyses and Consequences

Using whole genome sequence data, it was possible to detect and analyze historic trends in effective population size and access methods not available for genetic data sets. Most notably, the recent anthropogenic population bottleneck between the 1950s and 1970s was clearly evidenced (using GONE), with a sharp decrease and subsequent increase in *N*_e_ occurring concurrently across southern England (based on samples distributed across southwest, central, and eastern England). In contrast, Wales showed a decline beginning around 1830s, coinciding with the “improvement of the sporting gun with percussion detonation,” and reduced otter hunt results in North Wales (Jefferies 1989). *N*_e_ estimates from Wales increased from the 1950s (as other British populations were beginning to decline), which was unexpected but potentially due to the impact and changes of otter hunting around this time, despite legal protection only being obtained in 1978 in Wales and England (Jefferies 1989). The North showed an overall, gradual decline from around 1200 to the present day, with no change in this trend through the 1950s–1970s bottleneck detected in southern England, confirming suspicions that this larger, more rural population was less affected by chemical pollution (Chanin and Jefferies 1978). These findings are broadly in keeping with otter hunting records which, for example, indicate a decline in success rate of otter hunts from 1957 across southwest England, a lesser decline in northern England and Scotland, and no evidence of a decline in north Wales (Chanin and Jefferies 1978). The accompanying PSMC analyses also show a more long-term decline from 1,000,000 to 10,000 years ago across all populations.

We hypothesized that genomic signatures of a population bottleneck would be evident. Breeding between relatives leads to homozygous regions of the genome separately inherited from a recent shared ancestor, known as ROH. Longer ROH indicate recent inbreeding, and shorter runs indicate older inbreeding, which have been broken up by recombination (Druet and Gautier 2017). We predicted that the British bottleneck occurred between the 1950s and 1970s and would therefore be identifiable as ROH of a length corresponding to inbreeding occurring 9–17 generations before these samples were collected (based on a generation time of 4 years); however, ROH of this length were infrequent. ROH have been identified in populations suffering severe reductions in effective population size for prolonged periods of time, such as the Florida panther, *Puma concolor* (Saremi et al. 2019). We do not have census data spanning the bottleneck, but due to the banning of some chemical contaminants in the 1970s (Walker and Newton 1998), we predicted that the bottleneck was unlikely to have persisted for more than 20 years. However, the GONE analyses indicated that depending on the population, *N*_e_ dropped for between 30 and 45 years before increasing (with the exception of Wales and the North). Despite the length and severity of this bottleneck, especially in the East (from the lowest *N*_e_ estimate of around 100 to around 3), it does not appear to have led to a consistent burden of ROH across multiple individuals. Conversely, we observe many, short ROH across all samples. Theory would suggest this indicates very old inbreeding followed by generations of recombination, potentially from a bottleneck that is older than we are able to estimate using RzooROH or due to background relatedness (Ceballos et al. 2018). We found no evidence for bottlenecks from 10,000 to 1,000,000 years ago (PSMC analyses) nor from 200 to 800 years ago (GONE analyses); however, we have not assessed evidence for the period 800 to 10,000 years ago due to method limitations. It is unexpected, but reassuring, that we do not see long ROH in these individuals despite the severe recent bottleneck in the East. However, the evidence for extensive historic inbreeding and its legacy, the generally high realized inbreeding coefficient (*F*_ROH_) observed in the modern populations, provides cause for concern with respect to the species’ long-term viability, particularly given the current context of small populations and anthropogenic threats for a near threatened species (Roos et al. 2015; Reid et al. 2019).

### Distinct Signatures Detected in the Eastern Population

Unexpected diversity identified in the East using genomic methods was not previously identified in studies using genetic methods (Stanton et al. 2014). Estimates of variation (e.g., from PCA) were dominated by variation among samples within the East, and these results were matched by higher heterozygosity, nucleotide diversity, and private SNP counts, alongside lower inbreeding coefficients relative to other British populations. The GONE analyses indicate that the population from the East went through the most severe bottleneck of all British populations, with *N*_e_ as low as 3.7, making the high variation in this region particularly surprising. Although the ROH analyses identified some recent inbreeding, it was not consistent across individuals from the East, indicating a more complex demographic history than hypothesized from a simple anthropogenic bottleneck. Other metrics are also consistent with a relatively complex demographic history in the East population, including ADMIXTURE results, which indicate inconsistent population clustering, and fineSTRUCTURE results, which indicate that the East have lower shared coancestry with remaining populations than they show when compared with one another but also show inconsistent coancestry between individuals from the East. For example, although some pairs within the East show very high shared coancestry, others show very low shared coancestry, whereas all individuals in Wales show a very similar shared coancestry between pairs. Lastly, the divergent mitochondrial lineage identified using genomic methods was found only in samples from the East and the reference genome (from Somerset, equidistant to southwest and eastern samples used in this study). In line with previous studies (Thomas et al. 2022), SNP-based measures of pairwise *F*_ST_ were not larger for pairs including the East, suggesting that although the East contains lots of unique variation, it is not more distantly related to remaining populations. Since pairwise *F*_ST_ is a relative measure of genetic variation within a population relative to total variation, it is possible that these results reflect the high variation observed within the East, when compared with total variation.

The results observed in the East do not align with the simple scenario of a population bottleneck and recovery, and instead, we propose alternative hypotheses to explain our findings. In the first National Otter Survey of England, in 1977–1979, of 623 sites surveyed in East Anglia, only 20 were positive, illustrating how close the population was to local extinction (Lenton et al. 1980). Captive-bred otters were released, 13 in the south and 81 in the east of England, as a part of a broader reintroduction program of over 180 otters across Britain (Green 1997). Following these releases, the second National Survey in 1984–1986 found 8 positive out of 725 surveyed sites in East Anglia, 5 of which were from released otters (Strachan et al. 1990). One explanation for genetic distinctiveness of otters in the East might be that their small effective population size led to strong genetic drift. However, the high genetic diversity (both overall variability and the high private SNP count) observed in the genomic data in the East renders this explanation unlikely. Another explanation is that the East gene pool contains ancestry from Eurasian otters of non-British origin, explaining both the unusually high proportion of unique variation and the divergent mitochondrial lineage. A study by the European Association of Zoos and Aquaria (EAZA) using microsatellites identified two main genetic “lines” of Eurasian otters in captivity, known as A- and B-lines (E. Rey pers. comm.), where anecdotal evidence suggests the B-line otters were bred with the Asian subspecies *L. l. barang* found in Sumatra, Thailand, and Vietnam (J. Palmer pers. comm. (Hung and Law 2016)). An organization involved in the reintroduction program was separately breeding Eurasian otters in captivity using two founders from Thailand, thought at the time to be *L. l. barang* (J. Palmer pers. comm.). Although studbook records show no evidence of crossing between otters of Thai origin and British *L. lutra* (J. Palmer pers. comm.), escapes or unknown mixing between pens cannot be excluded, however unlikely (P. Chanin pers. comm.). Although there are no whole mitochondrial genome sequences available from Eurasian otters from Thailand, we have included a sequence from the neighboring Laos in this study, which groups with samples from China and the east of England. Therefore, when combined with our genomic results, these findings indicate the possibility of individuals of Asian origin (likely Thailand) being either accidentally bred, released, or escaping into the south and east of England. Otter releases in this region occurred between 1983 and 1996, coinciding with the increase in our estimate of effective population size for the East. We therefore suggest that the introduction of a few Asian-origin or admixed otters to a very small existing population had a large impact on the genetic identity of the population, leading to the high proportion of unique genomic variation and divergent mitochondrial lineages. The captive B-line otters are no longer being bred in captivity (J. Palmer pers. comm.); however, there are indications that some of their descendants have been released in Europe (Hájková et al. 2007). Our results show that the consequences of such introductions have left genomic signatures across the east of England and possibly beyond.

To provide further evidence describing the genetic history of otters in Britain, we suggest two main paths of future work. Firstly, to further investigate the history of otters in the East of Britain, it would be beneficial to sequence historical samples from the region (taken before the population bottleneck), as well as B-line captive-bred otters, to compare these to the population we observe presently and assess the likelihood of a replacement event occurring through the reintroductions (Strachan et al. 1990). Secondly, we recommend sampling the range of the Eurasian otter broadly, including historic samples, to try to identify any similarities between a potential source population for British reintroductions, such as *L. l. barang*, *L. l. chinensis* (found in Thailand), and other Asian lineages, alongside any other populations where B-line otters may have been reintroduced. This would also enable a thorough genomic assessment of subspecies classifications, clarifying the existing confusion around Asian subspecies, such as *L. l. barang* and *L. l. chinensis*. We note that population declines and local extinctions across the species’ range mean that it is possible that the source population could be missed in this sampling, highlighting the importance of historic samples (Yoxon and Yoxon 2019).

### Conservation Implications

Our genetic to genomic comparison highlights the value of genomic methods in conservation to avoid misinterpretations of potentially biased, low-resolution markers. However, this does not make the Eurasian otter microsatellite panel or previous studies based on this redundant. Rather, this study identifies the limits of the interpretations of these results when compared with genomic data. A SNP array for capture-based SNP genotyping could be designed based on our findings, to provide cheaper genomic analyses of British otters. However, based on our findings of ascertainment bias, we would recommend a SNP array based on SNPs identified from a range-wide sampling of the species.

Here, we provide the first clear evidence of a population bottleneck in Eurasian otters in southern England, highlighting an important contrast to the history of populations in northern England, Scotland, and Wales. We provide clear evidence of previously unexpected and unusual signatures in the east of England and assign these to a mitochondrial lineage only found, as yet, in Asia. Both of these findings have only been possible through the application of genomic methods, enabled by the prior publication of an extremely high-quality reference genome (Mead et al. 2020), illustrating the importance of WGS for conservation of this and other species.

## Methods

### Sample Collection and DNA Extraction

Samples from across the four known stronghold populations (the North of England and Scotland, Southwest England, Wales, and central and eastern England) were selected based on location from the Cardiff University Otter Project (CUOP) archive. The 45 individuals analyzed in this study were collected between 2016 and 2020 and comprised 35 adult males, 5 females (1–2 per stronghold), and 5 male juveniles (see supplementary material S1, Supplementary Material online). Genomic DNA was extracted from muscle tissue using a salt extraction protocol (Rivero et al. 2006) and stored in TLE buffer (1 M Tris, 0.5 M ethylenediaminetetraacetic acid [EDTA], pH 8). DNA quantity and quality were assessed using gel electrophoresis, and final concentrations ranged from 7.3 to 117.4 ng/µL.

### Whole Genome Sequencing

Sequencing was conducted as part of the Darwin Tree of Life (DToL) program (Blaxter et al. 2022) by the Wellcome Sanger Institute (Hinxton, UK). Whole genomes of 45 Eurasian otters were sequenced on the Illumina NovaSeq platform, with 150 bp paired-end reads. The standard DToL pipelines were used to quality filter, trim, and map reads to the Eurasian otter reference genome assembly (Mead et al. 2020). Variants were called using DeepVariant (Poplin et al. 2018), and joint variant calling was performed using GLNexus (Lin et al. 2018).

Unplaced scaffolds (*n* = 23) make up 0.34% of the total reference genome assembly and were discarded from all further analyses. Only biallelic SNPs from chromosome 1 to 18 (i.e., excluding X, Y, and mitochondrial scaffolds) were used in population genomic analyses, unless otherwise stated. Vcf subsetting, viewing, variant counting, indexing, and converting between file formats was conducted using BCFTools v1.14 (Danecek et al. 2021), and data handling and visualization was conducted in R version 4.2.0 (R Core Team 2019), using RStudio version 2022.2.3.492 (RStudio Team 2022) and the tidyverse packages (Wickham et al. 2019) unless otherwise stated.

### Microsatellite Genotyping

Genomic locations of microsatellite loci commonly used in studies of Eurasian otters were identified in the reference genome using Blast (see supplementary material S2.4, Supplementary Material online, for methods and results). Microsatellite genotyping was carried out for all 45 samples using 15 loci amplified in 3 multiplex PCR reactions (supplementary material S1, Supplementary Material online). PCR conditions were as in Hobbs et al. (2006) (supplementary material S2.3, Supplementary Material online). Fragment analysis on an ABI3730 capillary sequencer (Applied Biosystems) was conducted at MRC PPU DNA Sequencing and Services, Dundee, Scotland, using Rox400HD as a size marker. Microsatellite alleles were scored using the Microsatellite plugin in Geneious Prime v2022.0.2 (https://www.geneious.com). Four samples were replicated for all 3 multiplexes to ensure consistency, and 38 sample/multiplex combinations were rerun. One locus (Lut733) for one sample (mLutLut40) could not be genotyped reliably, providing inconsistent results across four replicates, resulting in an overall success rate of 99.85%.

### Population Structure

For the whole genome SNP data, PCAs were conducted in PLINK v1.9 (Purcell et al. 2007). Weir and Cockerham's (1984)  *F*_ST_ was calculated using VCFtools v0.1.16 (Danecek et al. 2011). A paired *t*-test was used to compare the differences in *F*_ST_ values between WGS and microsatellite data sets. STRUCTURE could not be run on this genomic data set due to computational limitations of the STRUCTURE algorithm when handling genomic-scale data sets, and therefore, ADMIXTURE was selected as the most algorithmically similar software. ADMIXTURE v1.3.0 (Alexander and Lange 2011) was run on the full SNP data set for *K* values of 1 to 8, and CV error was used to compare all *K* values. Details and results of the fineSTRUCTURE v4.1.1 (Lawson et al. 2012) and relatedness analyses are in supplementary materials S2.6 and S2.7, Supplementary Material online.

The microsatellite data was converted to Genepop format using MSA v4.05 (Dieringer and Schlötterer 2003), and when assessed using Genepop v4.6 (Raymond and Rousset 1995; Rousset 2008), no significant LD was detected in any pairwise comparisons among loci, even when compared across populations and after applying sequential Bonferroni correction (correcting the nominal *P* = 0.05 for multiple testing). All 15 loci were therefore kept for downstream analyses. Calculation of means and standard errors by population, of observed and expected microsatellite heterozygosity (*H*_O_, *H*_E_) and allele number (A), alongside PCA was conducted using the R package adegenet version 2.1.5 (Jombart 2008). Private microsatellite alleles were determined using the R package poppr version 2.9.4 (Kamvar et al. 2014). Inbreeding coefficient, *F*_IS_, and pairwise *F*_ST_ values were calculated using hierfstat version 0.5-10 (Goudet 2005). PopGenReport version 3.0.7 (Gruber and Adamack 2015) was used to calculate mean and standard errors of allelic richness by population. STRUCTURE v2.3.4 (Pritchard et al. 2000) was used to assess population structuring and admixture proportions. Each run consisted of a burn-in of 100,000, followed by 900,000 recorded iterations, run from *K* of 1 to 10, each repeated 10 times, using the admixture model (without sampling locations as priors) and assuming allele frequencies to be correlated. The ten repeated runs for each *K* value were then assessed for alternative solutions and combined using the CLUMPAK web server (Raymond and Rousset 1995; Dieringer and Schlötterer 2003; Rousset 2008; Kopelman et al. 2015).

### Genomic Diversity

Nucleotide diversity was calculated using VCFtools, in 20 Mb windows across all samples within each geographic population, alongside individual heterozygosity. SNPs private to a population, or found across any combination of populations, were counted using the vcf-compare tool within VCFtools. Due to the uneven sample sizes of the geographic populations, eight samples were randomly selected from each population to use for this analysis. Significant differences in genetic diversity measures among populations were assessed using ANOVA conducted in R. LD was calculated in VCFtools, for each geographic population, by chromosome, between all pairs of SNPs up to 100 kbp apart and averaged for each distance between SNPs before plotting.

### Runs of Homozygosity

Due to the ability of a single incorrectly called variant to break a ROH, variants were filtered separately for these analyses (supplementary material S2.12, Supplementary Material online). For ROH identification, we used RZooRoH v0.3.1 (Druet and Gautier 2017; Bertrand et al. 2019) to model homozygous-by-descent (HBD) segments of the genome, where the length corresponds to the number of generations since the common ancestor of the haplotype. Following model selection using the Bayesian Information Criterion, we used HBD segments from 4, 8, 16, 32, 64, 128, 256, 512, and 1,024 generations ago, alongside identifying non-HBD segments.

### Historic *N*_e_

We used GONE (Santiago et al. 2020) to estimate recent effective population size (*N*_e_) for each population (see supplementary material S2.9, Supplementary Material online, for full method). GONE calculates LD between pairs of SNPs over a range of recombination rates and finds the series of *N*_e_ that best explains the observed LD spectrum (Santiago et al. 2020). We ran all simulations for 2,000 generations calculated in 400 bins but only present results for the most recent 200 generations. We repeated each run 10 times, with each run taking a new subsample of 100,000 SNPs (comparable to bootstrapping), to incorporate variance in *N*_e_ estimates across runs. Recent estimates of *N*_e_ using GONE were compared with survey data of England, Wales, and Scotland from 1977 to 2010; details of these methods are in supplementary material S2.10, Supplementary Material online, and data in supplementary material S1, Supplementary Material online. PSMC inference (Li and Durbin 2011) was also used to estimate older effective population size (*N*_e_) changes (supplementary material S2.9, Supplementary Material online). A generation time of 4 years was used for GONE, PSMC, and ROH analyses (see supplementary material S2.9, Supplementary Material online).

### Mitochondrial Genome Analyses

We assembled the mitochondrial genome of each sample independently and combined these with previously published data and sequences (see supplementary material S2.11, Supplementary Material online, for full methods). NOVOPlasty v4.3.1 (Dierckxsens et al. 2017) was used to assemble the mitochondrial genomes from the adapter-trimmed reads using the 16,536 bp reference genome mitochondrial scaffold (LR822067.1) as the seed. NOVOPlasty failed to assemble four samples, of which MITObim v1.9.1 (Hahn et al. 2013) was used to assemble three samples.

We aimed to exclude the possibility that our results are assembled from nuclear sequences of mitochondrial origin (NUMTs) rather than true mitochondrial sequences, therefore biasing phylogenetic interpretation (Lucas et al. 2022). Despite no prior evidence of NUMTs in any mustelid species, NumtFinder (Edwards 2021) identified reasonably long putative NUMTs (from 5 to 9 kbp) across chromosomal and unplaced scaffolds in the Eurasian otter reference genome (mLutLut1.2). However, it was not possible to create the combination of variants identified in either whole mitochondrial genome lineage from any combination of NUMTs we identified. When all reads from one sample were mapped only to a mitochondrial genome sequence and sites differentiating between the two divergent lineages in this study were interrogated, we found that across ten randomly chosen sites, on average <1% of reads did not support the base assembled in the mitochondrial genome. Therefore, although we have identified the presence of NUMTs in *L. lutra*, we do not believe they are contributing to the divergent mitochondrial lineages identified in this study and instead conclude that we have assembled the true mitochondrial lineages (see supplementary material S2.11.1, Supplementary Material online).

Alongside the British samples, paired-end short-read data of samples from Russia (*n* = 1), Norway (*n* = 1), and Denmark (*n* = 1) were downloaded from the Sequence Read Archive (SRA) and assembled using NOVOPlasty as described above (table 1). Previously published mitochondrial sequences available from GenBank (*n* = 13, including the reference genome) were also incorporated into the analyses, alongside a hairy-nosed otter (*L. sumatrana*, KY117556) sequence as an outgroup (table 1).

Geneious Prime was used to align sequences using the MUSCLE algorithm (Edgar 2004), and due to uncertain repeat numbers surrounding the tandem repeat, this region was removed (positions 16,050–16,202 on the reference scaffold), leaving a total alignment length of 16,365 bp. To contextualize our samples with prior studies, 255 bp of the CR were extracted and compared with previously published CR haplotypes. PopArt v1.7 (Leigh and Bryant 2015) was used to produce statistical parsimony networks based on the TCS algorithm (Clement et al. 2002) and to identify the number of mutation steps between haplotypes.

After aligning to previously published *L. lutra* and outgroup whole mitochondrial genome sequences, the repeat region was again removed (positions 16,035–16,289 bp relative to the reference scaffold), leaving a total alignment length of 16,392 bp. The R packages pegas (Paradis 2010) and ape (Paradis and Schliep 2019) were used to calculate summary statistics (haplotype richness, haplotype diversity, and nucleotide diversity, π). IQ-TREE (Nguyen et al. 2015) ModelFinder (Kalyaanamoorthy et al. 2017) was used to identify the best fitting model based on the Bayesian Information Criterion, which was the three-substitution type model with unequal, empirical base frequencies (Kimura 1981), allowing for a proportion of invariable sites (‘K3Pu + F + I’). A consensus tree was constructed based on 1,000 bootstrap replicates (Hoang et al. 2018) and visualized using FigTree v1.4.4 (http://tree.bio.ed.ac.uk/software/figtree/).

## Supplementary Material

msad207_Supplementary_Data

## Data Availability

All raw data have been submitted to the International Nucleotide Sequence Database Collaboration (INSDC) archives and are available through the DToL Data Portal (https://portal.darwintreeoflife.org/data/root/details/Lutra%20lutra). Individual microsatellite genotype data are available in supplementary material S1, Supplementary Material online. Newly generated unique haplotype data are deposited to NCBI Nucleotide Database (OR633269-86 and BK064833-BK064835), and all whole mitochondrial genome sequences analysed in this study can be found on GitHub (https://github.com/sduplessis1/EurasianOtter_PopGen) alongside the bioinformatic code used for all analyses.
