# Supplementary material for: Genomics Reveals Complex Population History and Unexpected Diversity of Eurasian Otters (Lutra lutra) in Britain Relative to Genetic Methods
Source: Mol Biol Evol. 2023 Sep 15;40(11):msad207. doi: 10.1093/molbev/msad207 (PMC10630326; doi:10.1093/molbev/msad207)
Supplement: msad207_Supplementary_Data [file msad207_supplementary_data.zip › EurasianOtter_PopGen.html]

UK\_bioinformatics\_tobeshared.md


# Bioinformatic analyses for Eurasian otter population genomics

Scripts used to conduct bioinformatic analyses for manuscript (in review):
'Genomics reveals complex population history and unexpected diversity of Euraisan otters (*Lutra lutra*) in Britain relative to genetic methods, du Plessis *et al.*'

Briefly, we used whole genome short read sequencing of 45 Eurasian otters from across Britain to study the population genomics across 4 'stronghold' populations of East, North, Wales and Southwest England.

## Summary of wget.sh

Aim: Download vcf, rename samples, and select only autosomes and bialleleic SNPs for analyses.

```
#!/bin/bash
module load bcftools/1.14

## DOWNLOAD FILES ##
wget https://tolqc.cog.sanger.ac.uk/25g/mammals/Lutra_lutra/analysis/mLutLut1.2/variant/wgs/mLutLut.bcf.gz
wget https://tolqc.cog.sanger.ac.uk/25g/mammals/Lutra_lutra/analysis/mLutLut1.2/variant/wgs/mLutLut.bcf.gz.csi
# copied sumcheck into this doc, to check if files fully downloaded
md5sum --check sanger_sumcheck

## LINECOUNT ##
bcftools view -H mLutLut.bcf.gz | wc -l > mLutLut.bcf.gz_line

## RENAME SAMPLES ##
# Correcting sample names
bcftools reheader mLutLut.bcf.gz -s samples_names -o mLutLut_renamed.bcf.gz
bcftools index mLutLut_renamed.bcf.gz

## SELECT AUTOSOMES ##
# Select only chromosome level scaffolds (chr1-18)
bcftools view --regions-file GCA_902655055.2_mLutLut1.2_autosomes.bed mLutLut_renamed.bcf.gz -Oz -o mLutLut_renamed_autosomes.vcf.gz
bcftools index mLutLut_renamed_autosomes.vcf.gz
bcftools view -H mLutLut_renamed_autosomes.vcf.gz | wc -l > mLutLut_renamed_autosomes.vcf.gz_lines

## VARIANT TYPE ##
bcftools stats mLutLut_renamed_autosomes.vcf.gz > mLutLut_renamed_autosomes.vcf.gz_stats
# selecting only snps (-v snps), which are biallelic (-m 2 and -M 2)
bcftools view -v snps -m 2 -M 2 mLutLut_renamed_autosomes.vcf.gz -Oz -o mLutLut_renamed_autosomes_bisnps.vcf.gz
bcftools index mLutLut_renamed_autosomes_bisnps.vcf.gz
bcftools view -H mLutLut_renamed_autosomes_bisnps.vcf.gz | wc -l > mLutLut_renamed_autosomes_bisnps.vcf.gz_lines
```

## Summary of samples\_names

Aim: Samples names used as an input file for renaming samples (in wget.sh script).

```
mLutLut12	mLutLut27
mLutLut13	mLutLut39
mLutLut14	mLutLut51
mLutLut15	mLutLut63
mLutLut16	mLutLut75
mLutLut17	mLutLut87
mLutLut23	mLutLut64
mLutLut25	mLutLut88
mLutLut26	mLutLut5
mLutLut28	mLutLut29
mLutLut31	mLutLut77
mLutLut33	mLutLut89
mLutLut34	mLutLut6
mLutLut37	mLutLut42
mLutLut38	mLutLut54
mLutLut40	mLutLut78
mLutLut41	mLutLut90
mLutLut42	mLutLut7
mLutLut43	mLutLut19
mLutLut45	mLutLut43
mLutLut47	mLutLut67
mLutLut48	mLutLut79
mLutLut55	mLutLut68
mLutLut57	mLutLut92
mLutLut58	mLutLut9
mLutLut59	mLutLut21
mLutLut62	mLutLut57
mLutLut64	mLutLut81
mLutLut65	mLutLut93
mLutLut68	mLutLut34
mLutLut7	mLutLut62
mLutLut70	mLutLut58
mLutLut71	mLutLut70
mLutLut72	mLutLut82
mLutLut73	mLutLut94
mLutLut77	mLutLut47
mLutLut78	mLutLut59
mLutLut8	mLutLut74
mLutLut80	mLutLut83
mLutLut81	mLutLut95
mLutLut84	mLutLut36
mLutLut87	mLutLut72
mLutLut9	mLutLut86
mLutLut91	mLutLut25
mLutLut96	mLutLut85
```

## Summary of GCA\_902655055.2\_mLutLut1.2\_autosomes.bed

Aim: Bed file defining the autosomes for further analyses (used as input for wget.sh script).

```
LR738403.1      0       223449203
LR738404.1      0       210645437
LR738405.1      0       201318998
LR738406.1      0       197708286
LR738407.1      0       165805745
LR738408.1      0       154425511
LR738409.1      0       149004807
LR738410.1      0       144745594
LR738411.1      0       144085464
LR738412.1      0       114662049
LR738413.1      0       108787771
LR738414.1      0       96445735
LR738415.1      0       95726014
LR738416.1      0       89080780
LR738417.1      0       69992071
LR738418.1      0       61483029
LR738419.1      0       60348511
LR738420.1      0       40428123
```

## Summary of blastdb.sh

Aim: Build BLAST database of the otter reference genome, and search for microsatellite sequences.

```
#!/bin/bash
module load blast/2.12.0

## make blast database from reference genome ##
makeblastdb -in GCA_902655055.2_mLutLut1.2_genomic.fna \
	-parse_seqids -blastdb_version 5 -dbtype nucl

## blast sequences against the database ##
# give the output in 2 formats (-outfmt 6, and default)
blastn -db GCA_902655055.2_mLutLut1.2_genomic.fna \
    -query our_otter_msats.fsa -out our_otter_msats_out.txt
blastn -db GCA_902655055.2_mLutLut1.2_genomic.fna \
    -query our_otter_msats.fsa -out our_otter_msats_out_fmt.txt -outfmt 6
```

## Summary of admixturepca.sh

Aim: File prep (adding sample and population names to plink files), then running PCA and ADMIXTURE.

```
#!/bin/bash
module load vcftools/0.1.16
module load bcftools/1.14
PLINK=/path/to/PLINK/

## INPUTS ##
VCF_1=mLutLut_renamed_autosomes_bisnps.vcf.gz
VCF_2=mLutLut_renamed_autosomes_bisnps_chromcodes.vcf.gz
PLINK_OUT=mLutLut_renamed_autosomes_bisnps_chromcodes

## FILE PREP ##
#rename chromosomes to integers (for plink and admixture)
bcftools annotate $VCF_1 --rename-chrs chrom_names -o $VCF_2
# read into plink/make bed file, also asking plink to treat half call genotypes (0/.) as missing data
$PLINK --vcf $VCF_2 --make-bed --vcf-half-call missing --out $PLINK_OUT
# re-write ped file and make bed with populations and sex of samples
$PLINK --bfile $PLINK_OUT --update-ids pop_ids --make-bed --out $PLINK_OUT
$PLINK --bfile $PLINK_OUT --update-sex sex --make-bed --out $PLINK_OUT

## PCA ##
$PLINK --bfile $PLINK_OUT --pca --out $PLINK_OUT

## ADMIXTURE ##
# This loop runs admixture at a range of K values (1-8), threaded over 4 cpu (-j4), and using the default cross validation at 5-fold (--cv)
for K in `seq 1 8`;
do
	/path/to/admixture/admixture ${PLINK_OUT}.bed $K --cv -j4 ;
done
```

## Summary of gendiv\_fst.sh

Aim: Calculate FSTs between all population pairs.

```
#!/bin/bash
module load vcftools/0.1.16

VCF=mLutLut_renamed_autosomes_bisnps.vcf.gz

# calculates Fst between the pair of populations provided e.g. first command runs for east and north as a pair
vcftools --gzvcf $VCF --weir-fst-pop pop_east --weir-fst-pop pop_north --out mLutLut_pairwisefst_east_north
vcftools --gzvcf $VCF --weir-fst-pop pop_east --weir-fst-pop pop_wales --out mLutLut_pairwisefst_east_wales
vcftools --gzvcf $VCF --weir-fst-pop pop_east --weir-fst-pop pop_sweng --out mLutLut_pairwisefst_east_sweng
vcftools --gzvcf $VCF --weir-fst-pop pop_north --weir-fst-pop pop_wales --out mLutLut_pairwisefst_north_wales
vcftools --gzvcf $VCF --weir-fst-pop pop_north --weir-fst-pop pop_sweng --out mLutLut_pairwisefst_north_sweng
vcftools --gzvcf $VCF --weir-fst-pop pop_wales --weir-fst-pop pop_sweng --out mLutLut_pairwisefst_wales_sweng
```

## Summary of gendiv\_all.sh

Aim: Calculate heterozygosity, nucleotide diversity and relatedness. In the same file as this script are 4 files called pop\_east, pop\_wales, pop\_north and pop\_sweng, and each file contains a list of sample ids belonging to that population.

```
#!/bin/bash
module load vcftools/0.1.16

VCF=mLutLut_renamed_autosomes_bisnps.vcf.gz
OUT_ALL=mLutLut

# heterozygosity
vcftools --gzvcf $VCF --het --out $OUT_ALL

# relatedness
vcftools --gzvcf $VCF --relatedness2 --out mLutLut

#population loop
INDS=($(for i in pop*
do
       echo $(basename ${i})
done))

# calculate pi within each population
for IND in ${INDS[@]}
do
    OUT=mLutLut_${IND}
	vcftools --gzvcf $VCF --window-pi 20000000 --keep ${IND} --out ${OUT}_20mb
done
```

## Summary of parallel\_shapeit.sh

Aim: Sample phasing using shapeit, run in parallel across chromosomes.

```
#!/bin/bash
module load bcftools/1.14
module load htslib/1.10.2
module load parallel/20200522

# file names
VCF_IN=mLutLut_renamed_autosomes_bisnps.vcf.gz
VCF_NOMISS=mLutLut_renamed_autosomes_bisnps_nomiss.vcf.gz
export VCF_NOMISS
OUTPUT=mLutLut_renamed_autosomes_bisnps_nomiss_phased
export OUTPUT

# remove missing data and index
bcftools view -e 'GT[*] = "mis"' $VCF_IN -Oz -o $VCF_NOMISS
bcftools index $VCF_NOMISS

# Function to run shapeit for phasing (and index)
function shapeit_phasing {
    /path/to/shapeit4.2 --input $VCF_NOMISS \
        --output ${OUTPUT}.${1}.vcf.gz \
        --region $1 \
        --sequencing
	bcftools index ${OUTPUT}.${1}.vcf.gz
}
export -f shapeit_phasing

# Function for a list of chromosomes found in a vcf file
function vcf_chromosomes {
    bcftools query -f '%CHROM\n' $1 | uniq
}
export vcf_chromosomes

# Apply the function in parallel, split by chromosome, threaded over 32 cpu (-j 32)
chrom_set=`vcf_chromosomes $VCF_NOMISS`
parallel --verbose -j 32 shapeit_phasing ::: ${chrom_set}
```

## Summary of finestructure.sh

Aim: Running finestructure on the phased dataset.

```
#!/bin/bash
module load bcftools/1.14
module load perl/5.26.0
FINESTRUC=/path/to/fs_4.1.1

# list of sample ids
bcftools query -l ${VCF_PATH}/mLutLut_renamed_autosomes_bisnps_nomiss_phased.LR738403.1.vcf.gz > data.ids

# loop over each chromosome file
FILES=($(for i in ${VCF_PATH}/mLutLut_renamed_autosomes_bisnps_nomiss_phased.LR7384*.1.vcf.gz
do
        echo $(basename ${i%.vcf.gz})
done))

for FILE in ${FILES[@]}
do
	# converting format (back to shapeit format) and unzipping
	bcftools convert ${VCF_PATH}/${FILE}.vcf.gz --hapsample ${FILE}
	gunzip ${FILE}.hap.gz
	# on to chromopainter format
	eval $(perl -I /path/to/perl5 -Mlocal::lib)
	# converting from IMPUTE2 format to ChromoPainters PHASE and RECOMBFILES
	perl $FINESTRUC/impute2chromopainter.pl -J ${FILE}.hap ${FILE}
	# create recombination rate map for linkage model of chromopainter
	perl $FINESTRUC/makeuniformrecfile.pl ${FILE}.phase ${FILE}.recombfile

done

# Running finestructure pipeline
$FINESTRUC/fs_linux_glibc2.3 whole.cp -n \
	-phasefiles mLutLut_renamed_autosomes_bisnps_nomiss_phased.LR7384{03..20}.1.phase \
    -recombfiles mLutLut_renamed_autosomes_bisnps_nomiss_phased.LR7384{03..20}.1.recombfile \
    -idfile data.ids -go

# Running finestructure without recombination map
$FINESTRUC/fs_linux_glibc2.3 whole.cp -n \
        -phasefiles mLutLut_renamed_autosomes_bisnps_nomiss_phased.LR7384{03..20}.1.phase \
        -idfile data.ids -go
```

## Summary of privatesnps.sh

Aim: Calculating private SNPs from genomic data.

```
#!/bin/bash
module load bcftools/1.14
module load vcftools/0.1.16
module load htslib/1.10.2

VCF=mLutLut_renamed_autosomes_bisnps.vcf.gz

## 1 - Split main vcf into population level files ##
EAST=mLutLut_renamed_autosomes_bisnps_east.vcf.gz
SWENG=mLutLut_renamed_autosomes_bisnps_sweng.vcf.gz
NORTH=mLutLut_renamed_autosomes_bisnps_north.vcf.gz
WALES=mLutLut_renamed_autosomes_bisnps_wales.vcf.gz

bcftools view $VCF --samples-file pop_east -Oz -o $EAST
bcftools index $EAST

bcftools view $VCF --samples-file pop_sweng -Oz -o $SWENG
bcftools index $SWENG

bcftools view $VCF --samples-file pop_north -Oz -o $NORTH
bcftools index $NORTH

bcftools view $VCF --samples-file pop_wales -Oz -o $WALES
bcftools index $WALES

## 2 - Remove empty lines (so that the vcf only contains lines/variants found in that pop) ##
EAST1=mLutLut_renamed_autosomes_bisnps_east_noemptylines.vcf
SWENG1=mLutLut_renamed_autosomes_bisnps_sweng_noemptylines.vcf
NORTH1=mLutLut_renamed_autosomes_bisnps_north_noemptylines.vcf
WALES1=mLutLut_renamed_autosomes_bisnps_wales_noemptylines.vcf

vcf-subset -e $EAST > $EAST1
bgzip $EAST1
tabix -p vcf ${EAST1}.gz

vcf-subset -e $SWENG > $SWENG1
bgzip $SWENG1
tabix -p vcf ${SWENG1}.gz

vcf-subset -e $WALES > $WALES1
bgzip $WALES1
tabix -p vcf ${WALES1}.gz

vcf-subset -e $NORTH > $NORTH1
bgzip $NORTH1
tabix -p vcf ${NORTH1}.gz

## 3 - Use vcf-compare to compare snps found in any combination of input files ##
	# output written to jobid.out
vcf-compare ${EAST1}.gz ${SWENG1}.gz ${WALES1}.gz ${NORTH1}.gz
```

## Summary of gendiv\_ld.sh

Aim: Calculating linkage disequilibrium by population, in parallel.

```
#!/bin/bash
module load vcftools/0.1.16
module load parallel/20200522
module load bcftools/1.14

VCF=mLutLut_renamed_autosomes_bisnps.vcf.gz
export VCF

# loop of population files
INDS=($(for i in pop*
do
        echo $(basename ${i})
done))
export INDS

# function to list chromosomes found in vcf
function vcf_chromosomes {
    bcftools query -f '%CHROM\n' $1 | uniq
}
export vcf_chromosomes

# function to calculate linkage disequilbrium (--geno-r2) in 100kbp sliding window (--ld-window-bp 100000), always using the same $VCF input file
# $1 defines the samples to calculate ld across (i.e. only samples from 1 population)
# $2 deines the chromosome to calculate ld across (i.e. only 1 chromosome at a time)
function ld_bypop {
	vcftools --gzvcf $VCF \
		--chr ${2} \
		--keep ${1} \
		--out mLutLut_${1}_${2} \
		--geno-r2 \
		--ld-window-bp 100000
}
export -f ld_bypop

# loop over each population
# within each loop, ld is calculated in parallel across each chromosome
for FILE in ${INDS[@]}
do
	chrom_set=`vcf_chromosomes $VCF`
	parallel --verbose ld_bypop ${FILE} ::: ${chrom_set} 
done
```

## Summary of rzooroh.sh

Aim: File prep and filtering for running RzooROH.

```
#!/bin/bash

module load bcftools/1.14

VCF_IN=mLutLut_renamed_autosomes_bisnps.vcf.gz

# split the whole vcf into sample level files
bcftools +split $VCF_IN -Oz -o .

# filter by depth MIN=1/2 and MAX=2* average (see xls sheet)
# For future runs, there are much more efficient ways of coding this!!!
bcftools filter -S . -i 'FMT/DP>10 & FMT/DP<39' mLutLut19.vcf.gz -Oz -o mLutLut19_dp.vcf.gz
bcftools filter -S . -i 'FMT/DP>11 & FMT/DP<44' mLutLut21.vcf.gz -Oz -o mLutLut21_dp.vcf.gz
bcftools filter -S . -i 'FMT/DP>14 & FMT/DP<55' mLutLut25.vcf.gz -Oz -o mLutLut25_dp.vcf.gz
bcftools filter -S . -i 'FMT/DP>10 & FMT/DP<41' mLutLut27.vcf.gz -Oz -o mLutLut27_dp.vcf.gz
bcftools filter -S . -i 'FMT/DP>10 & FMT/DP<40' mLutLut29.vcf.gz -Oz -o mLutLut29_dp.vcf.gz
bcftools filter -S . -i 'FMT/DP>11 & FMT/DP<45' mLutLut34.vcf.gz -Oz -o mLutLut34_dp.vcf.gz
bcftools filter -S . -i 'FMT/DP>9 & FMT/DP<34' mLutLut36.vcf.gz -Oz -o mLutLut36_dp.vcf.gz
bcftools filter -S . -i 'FMT/DP>10 & FMT/DP<40' mLutLut39.vcf.gz -Oz -o mLutLut39_dp.vcf.gz
bcftools filter -S . -i 'FMT/DP>11 & FMT/DP<42' mLutLut42.vcf.gz -Oz -o mLutLut42_dp.vcf.gz
bcftools filter -S . -i 'FMT/DP>13 & FMT/DP<53' mLutLut43.vcf.gz -Oz -o mLutLut43_dp.vcf.gz
bcftools filter -S . -i 'FMT/DP>8 & FMT/DP<33' mLutLut47.vcf.gz -Oz -o mLutLut47_dp.vcf.gz
bcftools filter -S . -i 'FMT/DP>16 & FMT/DP<65' mLutLut5.vcf.gz -Oz -o mLutLut5_dp.vcf.gz
bcftools filter -S . -i 'FMT/DP>9 & FMT/DP<35' mLutLut51.vcf.gz -Oz -o mLutLut51_dp.vcf.gz
bcftools filter -S . -i 'FMT/DP>14 & FMT/DP<57' mLutLut54.vcf.gz -Oz -o mLutLut54_dp.vcf.gz
bcftools filter -S . -i 'FMT/DP>9 & FMT/DP<36' mLutLut57.vcf.gz -Oz -o mLutLut57_dp.vcf.gz
bcftools filter -S . -i 'FMT/DP>10 & FMT/DP<39' mLutLut58.vcf.gz -Oz -o mLutLut58_dp.vcf.gz
bcftools filter -S . -i 'FMT/DP>9 & FMT/DP<36' mLutLut59.vcf.gz -Oz -o mLutLut59_dp.vcf.gz
bcftools filter -S . -i 'FMT/DP>9 & FMT/DP<38' mLutLut6.vcf.gz -Oz -o mLutLut6_dp.vcf.gz
bcftools filter -S . -i 'FMT/DP>12 & FMT/DP<47' mLutLut62.vcf.gz -Oz -o mLutLut62_dp.vcf.gz
bcftools filter -S . -i 'FMT/DP>12 & FMT/DP<49' mLutLut63.vcf.gz -Oz -o mLutLut63_dp.vcf.gz
bcftools filter -S . -i 'FMT/DP>11 & FMT/DP<42' mLutLut64.vcf.gz -Oz -o mLutLut64_dp.vcf.gz
bcftools filter -S . -i 'FMT/DP>8 & FMT/DP<31' mLutLut67.vcf.gz -Oz -o mLutLut67_dp.vcf.gz
bcftools filter -S . -i 'FMT/DP>10 & FMT/DP<41' mLutLut68.vcf.gz -Oz -o mLutLut68_dp.vcf.gz
bcftools filter -S . -i 'FMT/DP>9 & FMT/DP<35' mLutLut7.vcf.gz -Oz -o mLutLut7_dp.vcf.gz
bcftools filter -S . -i 'FMT/DP>13 & FMT/DP<51' mLutLut70.vcf.gz -Oz -o mLutLut70_dp.vcf.gz
bcftools filter -S . -i 'FMT/DP>10 & FMT/DP<41' mLutLut72.vcf.gz -Oz -o mLutLut72_dp.vcf.gz
bcftools filter -S . -i 'FMT/DP>10 & FMT/DP<41' mLutLut74.vcf.gz -Oz -o mLutLut74_dp.vcf.gz
bcftools filter -S . -i 'FMT/DP>16 & FMT/DP<63' mLutLut75.vcf.gz -Oz -o mLutLut75_dp.vcf.gz
bcftools filter -S . -i 'FMT/DP>14 & FMT/DP<56' mLutLut77.vcf.gz -Oz -o mLutLut77_dp.vcf.gz
bcftools filter -S . -i 'FMT/DP>14 & FMT/DP<55' mLutLut78.vcf.gz -Oz -o mLutLut78_dp.vcf.gz
bcftools filter -S . -i 'FMT/DP>14 & FMT/DP<57' mLutLut79.vcf.gz -Oz -o mLutLut79_dp.vcf.gz
bcftools filter -S . -i 'FMT/DP>19 & FMT/DP<76' mLutLut81.vcf.gz -Oz -o mLutLut81_dp.vcf.gz
bcftools filter -S . -i 'FMT/DP>15 & FMT/DP<61' mLutLut82.vcf.gz -Oz -o mLutLut82_dp.vcf.gz
bcftools filter -S . -i 'FMT/DP>19 & FMT/DP<75' mLutLut83.vcf.gz -Oz -o mLutLut83_dp.vcf.gz
bcftools filter -S . -i 'FMT/DP>9 & FMT/DP<36' mLutLut85.vcf.gz -Oz -o mLutLut85_dp.vcf.gz
bcftools filter -S . -i 'FMT/DP>11 & FMT/DP<44' mLutLut86.vcf.gz -Oz -o mLutLut86_dp.vcf.gz
bcftools filter -S . -i 'FMT/DP>10 & FMT/DP<41' mLutLut87.vcf.gz -Oz -o mLutLut87_dp.vcf.gz
bcftools filter -S . -i 'FMT/DP>12 & FMT/DP<48' mLutLut88.vcf.gz -Oz -o mLutLut88_dp.vcf.gz
bcftools filter -S . -i 'FMT/DP>13 & FMT/DP<53' mLutLut89.vcf.gz -Oz -o mLutLut89_dp.vcf.gz
bcftools filter -S . -i 'FMT/DP>12 & FMT/DP<48' mLutLut9.vcf.gz -Oz -o mLutLut9_dp.vcf.gz
bcftools filter -S . -i 'FMT/DP>12 & FMT/DP<47' mLutLut90.vcf.gz -Oz -o mLutLut90_dp.vcf.gz
bcftools filter -S . -i 'FMT/DP>17 & FMT/DP<68' mLutLut92.vcf.gz -Oz -o mLutLut92_dp.vcf.gz
bcftools filter -S . -i 'FMT/DP>15 & FMT/DP<60' mLutLut93.vcf.gz -Oz -o mLutLut93_dp.vcf.gz
bcftools filter -S . -i 'FMT/DP>9 & FMT/DP<34' mLutLut94.vcf.gz -Oz -o mLutLut94_dp.vcf.gz
bcftools filter -S . -i 'FMT/DP>10 & FMT/DP<41' mLutLut95.vcf.gz -Oz -o mLutLut95_dp.vcf.gz

bcftools index mLutLut19_dp.vcf.gz
bcftools index mLutLut21_dp.vcf.gz
bcftools index mLutLut25_dp.vcf.gz
bcftools index mLutLut27_dp.vcf.gz
bcftools index mLutLut29_dp.vcf.gz
bcftools index mLutLut34_dp.vcf.gz
bcftools index mLutLut36_dp.vcf.gz
bcftools index mLutLut39_dp.vcf.gz
bcftools index mLutLut42_dp.vcf.gz
bcftools index mLutLut43_dp.vcf.gz
bcftools index mLutLut47_dp.vcf.gz
bcftools index mLutLut5_dp.vcf.gz
bcftools index mLutLut51_dp.vcf.gz
bcftools index mLutLut54_dp.vcf.gz
bcftools index mLutLut57_dp.vcf.gz
bcftools index mLutLut58_dp.vcf.gz
bcftools index mLutLut59_dp.vcf.gz
bcftools index mLutLut6_dp.vcf.gz
bcftools index mLutLut62_dp.vcf.gz
bcftools index mLutLut63_dp.vcf.gz
bcftools index mLutLut64_dp.vcf.gz
bcftools index mLutLut67_dp.vcf.gz
bcftools index mLutLut68_dp.vcf.gz
bcftools index mLutLut7_dp.vcf.gz
bcftools index mLutLut70_dp.vcf.gz
bcftools index mLutLut72_dp.vcf.gz
bcftools index mLutLut74_dp.vcf.gz
bcftools index mLutLut75_dp.vcf.gz
bcftools index mLutLut77_dp.vcf.gz
bcftools index mLutLut78_dp.vcf.gz
bcftools index mLutLut79_dp.vcf.gz
bcftools index mLutLut81_dp.vcf.gz
bcftools index mLutLut82_dp.vcf.gz
bcftools index mLutLut83_dp.vcf.gz
bcftools index mLutLut85_dp.vcf.gz
bcftools index mLutLut86_dp.vcf.gz
bcftools index mLutLut87_dp.vcf.gz
bcftools index mLutLut88_dp.vcf.gz
bcftools index mLutLut89_dp.vcf.gz
bcftools index mLutLut9_dp.vcf.gz
bcftools index mLutLut90_dp.vcf.gz
bcftools index mLutLut92_dp.vcf.gz
bcftools index mLutLut93_dp.vcf.gz
bcftools index mLutLut94_dp.vcf.gz
bcftools index mLutLut95_dp.vcf.gz

# merge all sample level files back togther
bcftools merge mLutLut*_dp.vcf.gz -Oz -o mLutLut_dp.vcf.gz

# filter quality to include only >30
QUAL=mLutLut_dp_q.vcf.gz
bcftools view -i 'QUAL>30' mLutLut_dp.vcf.gz -Oz -o $QUAL
bcftools index $QUAL
bcftools view -H $QUAL | wc -l > ${QUAL}_lines

## convert to oxford gen format
bcftools convert -s mLutLut63,mLutLut64,mLutLut78,mLutLut90,mLutLut19,mLutLut92,mLutLut21,mLutLut81,mLutLut93,mLutLut74,mLutLut83,mLutLut85 \
	-g east_whole --chrom --tag GT $QUAL

bcftools convert -s mLutLut27,mLutLut29,mLutLut43,mLutLut67,mLutLut79,mLutLut68,mLutLut34,mLutLut62,mLutLut70,mLutLut82,mLutLut95,mLutLut72,mLutLut86 \
	-g north_whole --chrom --tag GT $QUAL

bcftools convert -s mLutLut39,mLutLut5,mLutLut77,mLutLut6,mLutLut7,mLutLut9,mLutLut94,mLutLut36 \
	-g sweng_whole --chrom --tag GT $QUAL

bcftools convert -s mLutLut51,mLutLut75,mLutLut87,mLutLut88,mLutLut89,mLutLut42,mLutLut54,mLutLut57,mLutLut58,mLutLut47,mLutLut59,mLutLut25 \
	-g wales_whole --chrom --tag GT $QUAL
```

## Summary of rzooroh\_model.R

Aim: R script for the RzooROH model run for these analyses, and writing pdf plots.

```
#!/usr/bin/Rscript

##specify library path in R
.libPaths("/path/to/Rpackages")
library(RZooRoH)
library(R.utils)
library(dplyr)

#### READING IN DATA ####
east_in <- ("east_whole.gen.gz")
east_samples <- ("east_whole.samples")
east <- zoodata(genofile = east_in, zformat = "gp",
                 samplefile = east_samples)
rm(east_in, east_samples)

wales_in <- ("wales_whole.gen.gz")
wales_samples <- ("wales_whole.samples")
wales <- zoodata(genofile = wales_in, zformat = "gp",
                samplefile = wales_samples)
rm(wales_in, wales_samples)

sweng_in <- ("sweng_whole.gen.gz")
sweng_samples <- ("sweng_whole.samples")
sweng <- zoodata(genofile = sweng_in, zformat = "gp",
                samplefile = sweng_samples)
rm(sweng_in, sweng_samples)

north_in <- ("north_whole.gen.gz")
north_samples <- ("north_whole.samples")
north <- zoodata(genofile = north_in, zformat = "gp",
                samplefile = north_samples)
rm(north_in, north_samples)

#### Defining the model ####
model <- zoomodel(K=10, krates=c(8, 16, 32, 64, 128, 256, 512, 1024, 2048, 5120))

#### Running zoorun ####
wales_model <- zoorun(model, wales)
east_model <- zoorun(model, east)
sweng_model <- zoorun(model, sweng)
north_model <- zoorun(model, north)


#### Results ####
# use BIC to compare models
bic <- c(wales_model@modbic, north_model@modbic, east_model@modbic, sweng_model@modbic)
write.csv(bic, "bic_model.csv")

# plot proportion of genome associated with different HBD classes as a bar chart
pdf(file="plot1_model.pdf",height=6,width=10)
zooplot_prophbd(list(Wales=wales_model,North=north_model,
                     East=east_model, SWEng=sweng_model),style='barplot')
dev.off()

# plot average inbreeding coefficients, cumulatively
pdf(file="plot2_model.pdf",height=6,width=10)
zooplot_prophbd(list(Wales=wales_model,North=north_model,
                     East=east_model, SWEng=sweng_model),style='lines', cumulative = TRUE)
dev.off()

# plot each individuals inbreeding coefficient, cumulatively
pdf(file="plot3_model.pdf",height=12,width=12)
zooplot_individuals(list(Wales=wales_model,North=north_model,
                         East=east_model, SWEng=sweng_model), cumulative = T)
dev.off()

# plot bar chart of inbreeding coefficient by individual, colour coded/stacked by HBD class
pdf(file="plot4_model.pdf",height=6,width=10)
zooplot_partitioning(list(Wales=wales_model,North=north_model,
                          East=east_model, SWEng=sweng_model), nonhbd = FALSE,
                     col=c("yellow", "orange", "pink", "green", "purple", "blue", "red",
                           "brown", "cyan", "coral"))
dev.off()

# plots the segements/locations of runs across all samples e.g. from 0-40mbp of chromosome 1
pdf(file="plot5_model.pdf",height=12,width=12)
zooplot_hbdseg(list(Wales=wales_model,North=north_model,
                    East=east_model, SWEng=sweng_model), chr = 1, coord=c(0,40000000))
dev.off()

pdf(file="plot5a_model.pdf",height=12,width=12)
zooplot_hbdseg(list(Wales=wales_model,North=north_model,
                    East=east_model, SWEng=sweng_model), chr = 1, coord=c(0,10000000))
dev.off()

## Re-formatting model output, for saving as a csv
df1_V2 <- as.data.frame(cbind(wales_model@sampleids, wales_model@realized[,1]))
df1_V3 <- as.data.frame(cbind(wales_model@sampleids, wales_model@realized[,2]))
df1_V4 <- as.data.frame(cbind(wales_model@sampleids, wales_model@realized[,3]))
df1_V5 <- as.data.frame(cbind(wales_model@sampleids, wales_model@realized[,4]))
df1_V6 <- as.data.frame(cbind(wales_model@sampleids, wales_model@realized[,5]))
df1_V7 <- as.data.frame(cbind(wales_model@sampleids, wales_model@realized[,6]))
df1_V8 <- as.data.frame(cbind(wales_model@sampleids, wales_model@realized[,7]))
df1_V9 <- as.data.frame(cbind(wales_model@sampleids, wales_model@realized[,8]))
df1_V10 <- as.data.frame(cbind(wales_model@sampleids, wales_model@realized[,9]))
df1_V11 <- as.data.frame(cbind(wales_model@sampleids, wales_model@realized[,10]))

df1_V2$kclass <- "2"
df1_V3$kclass <- "4"
df1_V4$kclass <- "8"
df1_V5$kclass <- "16"
df1_V6$kclass <- "32"
df1_V7$kclass <- "64"
df1_V8$kclass <- "128"
df1_V9$kclass <- "256"
df1_V10$kclass <- "320"
df1_V11$kclass <- "non-HBD"

df1_wales <- rbind(df1_V2, df1_V3, df1_V4, df1_V5, df1_V6, df1_V7, df1_V8, df1_V9, df1_V10, df1_V11)
df1_wales$pop <- "Wales"
df1_wales$v2 <- as.numeric(df1_wales$V2)
df1_wales$kclass <- as.factor(df1_wales$kclass)
summary(df1_wales)


df1_V2 <- as.data.frame(cbind(east_model@sampleids, east_model@realized[,1]))
df1_V3 <- as.data.frame(cbind(east_model@sampleids, east_model@realized[,2]))
df1_V4 <- as.data.frame(cbind(east_model@sampleids, east_model@realized[,3]))
df1_V5 <- as.data.frame(cbind(east_model@sampleids, east_model@realized[,4]))
df1_V6 <- as.data.frame(cbind(east_model@sampleids, east_model@realized[,5]))
df1_V7 <- as.data.frame(cbind(east_model@sampleids, east_model@realized[,6]))
df1_V8 <- as.data.frame(cbind(east_model@sampleids, east_model@realized[,7]))
df1_V9 <- as.data.frame(cbind(east_model@sampleids, east_model@realized[,8]))
df1_V10 <- as.data.frame(cbind(east_model@sampleids, east_model@realized[,9]))
df1_V11 <- as.data.frame(cbind(east_model@sampleids, east_model@realized[,10]))

df1_V2$kclass <- "2"
df1_V3$kclass <- "4"
df1_V4$kclass <- "8"
df1_V5$kclass <- "16"
df1_V6$kclass <- "32"
df1_V7$kclass <- "64"
df1_V8$kclass <- "128"
df1_V9$kclass <- "256"
df1_V10$kclass <- "320"
df1_V11$kclass <- "non-HBD"

df1_east <- rbind(df1_V2, df1_V3, df1_V4, df1_V5, df1_V6, df1_V7, df1_V8, df1_V9, df1_V10, df1_V11)
df1_east$pop <- "east"
df1_east$v2 <- as.numeric(df1_east$V2)
df1_east$kclass <- as.factor(df1_east$kclass)
summary(df1_east)


df1_V2 <- as.data.frame(cbind(north_model@sampleids, north_model@realized[,1]))
df1_V3 <- as.data.frame(cbind(north_model@sampleids, north_model@realized[,2]))
df1_V4 <- as.data.frame(cbind(north_model@sampleids, north_model@realized[,3]))
df1_V5 <- as.data.frame(cbind(north_model@sampleids, north_model@realized[,4]))
df1_V6 <- as.data.frame(cbind(north_model@sampleids, north_model@realized[,5]))
df1_V7 <- as.data.frame(cbind(north_model@sampleids, north_model@realized[,6]))
df1_V8 <- as.data.frame(cbind(north_model@sampleids, north_model@realized[,7]))
df1_V9 <- as.data.frame(cbind(north_model@sampleids, north_model@realized[,8]))
df1_V10 <- as.data.frame(cbind(north_model@sampleids, north_model@realized[,9]))
df1_V11 <- as.data.frame(cbind(north_model@sampleids, north_model@realized[,10]))

df1_V2$kclass <- "2"
df1_V3$kclass <- "4"
df1_V4$kclass <- "8"
df1_V5$kclass <- "16"
df1_V6$kclass <- "32"
df1_V7$kclass <- "64"
df1_V8$kclass <- "128"
df1_V9$kclass <- "256"
df1_V10$kclass <- "320"
df1_V11$kclass <- "non-HBD"

df1_north <- rbind(df1_V2, df1_V3, df1_V4, df1_V5, df1_V6, df1_V7, df1_V8, df1_V9, df1_V10, df1_V11)
df1_north$pop <- "north"
df1_north$v2 <- as.numeric(df1_north$V2)
df1_north$kclass <- as.factor(df1_north$kclass)
summary(df1_north)


df1_V2 <- as.data.frame(cbind(sweng_model@sampleids, sweng_model@realized[,1]))
df1_V3 <- as.data.frame(cbind(sweng_model@sampleids, sweng_model@realized[,2]))
df1_V4 <- as.data.frame(cbind(sweng_model@sampleids, sweng_model@realized[,3]))
df1_V5 <- as.data.frame(cbind(sweng_model@sampleids, sweng_model@realized[,4]))
df1_V6 <- as.data.frame(cbind(sweng_model@sampleids, sweng_model@realized[,5]))
df1_V7 <- as.data.frame(cbind(sweng_model@sampleids, sweng_model@realized[,6]))
df1_V8 <- as.data.frame(cbind(sweng_model@sampleids, sweng_model@realized[,7]))
df1_V9 <- as.data.frame(cbind(sweng_model@sampleids, sweng_model@realized[,8]))
df1_V10 <- as.data.frame(cbind(sweng_model@sampleids, sweng_model@realized[,9]))
df1_V11 <- as.data.frame(cbind(sweng_model@sampleids, sweng_model@realized[,10]))

df1_V2$kclass <- "2"
df1_V3$kclass <- "4"
df1_V4$kclass <- "8"
df1_V5$kclass <- "16"
df1_V6$kclass <- "32"
df1_V7$kclass <- "64"
df1_V8$kclass <- "128"
df1_V9$kclass <- "256"
df1_V10$kclass <- "320"
df1_V11$kclass <- "non-HBD"

df1_sweng <- rbind(df1_V2, df1_V3, df1_V4, df1_V5, df1_V6, df1_V7, df1_V8, df1_V9, df1_V10, df1_V11)
df1_sweng$pop <- "sweng"
df1_sweng$v2 <- as.numeric(df1_sweng$V2)
df1_sweng$kclass <- as.factor(df1_sweng$kclass)
summary(df1_sweng)

df1_all <- rbind(df1_wales, df1_east, df1_sweng, df1_north)
df1_all$id <- as.factor(df1_all$V1)
df1_all$pop <- as.factor(df1_all$pop)
df1_all$roh <- as.numeric(df1_all$V2)
towrite <- select(df1_all, id, pop, kclass, roh)
summary(towrite)
write.csv(towrite, "rzooroh_froh_model.csv")
```

## Summary of gone\_bootstrapped.sh

Aim: Script to GONE, and bootstrap (random repeated sub-sampling) replicates in loop.

```
#!/bin/bash

IND=($(for i in {0..9}
do
	echo "$i"
done))

# loop to make 10 files for each population, copy all the required files (input and gone files) and run gone within each of the population replicates
for INDS in ${IND[@]}
do
	pop=north
	mkdir ${pop}_${INDS}
	cp -r new_run/* ${pop}_${INDS}/
	cp data_in/${pop}* ${pop}_${INDS}/
	cd ${pop}_${INDS}
	sbatch slurm_script_GONE.sh ${pop}
	cd ..
done
```

## Summary of east\_0/slurm\_script\_GONE.sh

Aim: GONE specification, this is an example of run 0 for the East population. This was run using the command: `sbatch slurm_script_GONE.sh east`

```
#!/bin/bash
#script_GONE.sh

########################################################

#Check number of arguments
if [ $# -ne 1 ]  
then
	echo "Usage: $0 <FILE>" 
	exit 1
fi

### Set arguments

FILE=$1  ### Data file name for files .ped and .map

### Take input parameters from file INPUT_PARAMETERS_FILE

source INPUT_PARAMETERS_FILE

###################### FILES NEEDED ########################

### data.ped
### data.map

### EXECUTABLES FILES NEEDED IN DIRECTORY PROGRAMMES:

### MANAGE_CHROMOSOMES2
### LD_SNP_REAL3
### SUMM_REP_CHROM3
### GONE (needs gcc/7.2.0)
### GONEaverages
### GONEparallel.sh

################### Remove previous output files ##################

if [ -f "OUTPUT_$FILE" ]
then
rm OUTPUT_$FILE
fi

if [ -f "Ne_$FILE" ]
then
rm Ne_$FILE
fi

################### Create temporary directory ##################

if [ -d "TEMPORARY_FILES" ]
then
rm -r TEMPORARY_FILES
fi

mkdir TEMPORARY_FILES

################### Obtain sample size, number of chromosomes, number of SNPs ##################

cp $FILE.map data.map
cp $FILE.ped data.ped

tr '\t' ' ' < data.map > KK1
cut -d ' ' -f1 < KK1 > KK2

grep -w "" -c data.ped > NIND

tail -n 1 data.map | tr '\t' ' ' | cut -d ' ' -f1 > NCHR

SAM=$(grep -w "" -c $FILE.ped)

NCHR=$(tail -n 1 data.map | tr '\t' ' ' | cut -d ' ' -f1)

for((i=1;i<=$NCHR;i++))
do
grep -wc "$i" < KK2 > NCHR$i
done

if [ -f "SNP_CHROM" ]
then
rm SNP_CHROM
fi

for((i=1;i<=$NCHR;i++))
do
cat NCHR$i >> SNP_CHROM
done

rm KK*

################### Divide ped and map files into chromosomes ##################

echo "DIVIDE .ped AND .map FILES IN CHROMOSOMES"
echo "DIVIDE .ped AND .map FILES IN CHROMOSOMES" > timefile

num=$RANDOM
echo "$num" > seedfile

./PROGRAMMES/MANAGE_CHROMOSOMES2>>out<<@
-99
$maxNSNP
@

rm NCHR*
rm NIND
rm SNP_CHROM
###mv checkfile TEMPORARY_FILES/

################### LOOP CHROMOSOMES ##################
### Analysis of linkage disequilibrium in windows of genetic
### distances between pairs of SNPs for each chromosome

if [ $maxNCHROM != -99 ]
then
NCHR=$maxNCHROM
fi

echo "RUNNING ANALYSIS OF CHROMOSOMES ..."
echo "RUNNING ANALYSIS OF CHROMOSOMES" >> timefile

options_for_LD="$SAM $MAF $PHASE $NGEN $NBIN $ZERO $DIST $cMMb"

if [ $threads -eq -99 ]
then
threads=$(getconf _NPROCESSORS_ONLN)
fi

START=$(date +%s)

cp chromosome* TEMPORARY_FILES/

###### LD_SNP_REAL3 #######

### Obtains values of c, d2, etc. for pairs of SNPs in bins for each chromosome
for ((n=1; n<=$NCHR; n++)); do echo $n; done | xargs -I % -P $threads bash -c "./PROGRAMMES/LD_SNP_REAL3 % $options_for_LD"

END=$(date +%s)
DIFF=$(( $END - $START ))
echo "CHROMOSOME ANALYSES took $DIFF seconds"
echo "CHROMOSOME ANALYSES took $DIFF seconds" >> timefile

######################## SUMM_REP_CHROM3 #########################
### Combination of all data gathered from chromosomes into a single output file

### Adds results from all chromosomes

for ((n=1; n<=$NCHR; n++))
do
cat outfileLD$n >> CHROM
echo "CHROMOSOME $n" >> OUTPUT
sed '2,3d' outfileLD$n > temp
mv temp outfileLD$n
cat parameters$n >> OUTPUT
done

mv outfileLD* TEMPORARY_FILES/
rm parameters*

./PROGRAMMES/SUMM_REP_CHROM3>>out<<@
$NGEN	NGEN
$NBIN	NBIN
$NCHR	NCHR
@

mv chrom* TEMPORARY_FILES/

echo "TOTAL NUMBER OF SNPs" >> OUTPUT_$FILE
cat nsnp >> OUTPUT_$FILE
echo -e "\n" >> OUTPUT_$FILE
echo "HARDY-WEINBERG DEVIATION" >> OUTPUT_$FILE
cat outfileHWD >> OUTPUT_$FILE
echo -e "\n" >> OUTPUT_$FILE
cat OUTPUT >> OUTPUT_$FILE
echo -e "\n" >> OUTPUT_$FILE
echo "INPUT FOR GONE" >> OUTPUT_$FILE
echo -e "\n" >> OUTPUT_$FILE
cat outfileLD >> OUTPUT_$FILE

rm nsnp
rm OUTPUT
rm CHROM

############################# GONE.cpp ##########################
### Obtain estimates of temporal Ne from GONE

echo "Running GONE"
echo "Running GONE" >> timefile
START=$(date +%s)

./PROGRAMMES/GONEparallel.sh -hc $hc outfileLD $REPS

END=$(date +%s)
DIFF=$(( $END - $START ))
echo "GONE run took $DIFF seconds"
echo "GONE run took $DIFF seconds" >> timefile

echo "END OF ANALYSES"
echo "END OF ANALYSES" >> timefile

mv outfileLD_Ne_estimates Output_Ne_$FILE
mv outfileLD_d2_sample Output_d2_$FILE
rm outfileLD
rm data.ped
rm data.map
rm out
mv outfileLD_TEMP TEMPORARY_FILES/

###################################################################
```

## Summary of novoplasty.sh

Aim: Loop to run Novoplasty to assemble whole mitochondrial genomes on .cram files.

```
#!/bin/bash

module load perl/5.34.0
module load samtools/1.13
module load Trimmomatic/0.39
module load parallel/20200522

REF=GCA_902655055.2_mLutLut1.2_genomic.fna

# Make mitogenome ref scaffold for seed
grep -A 10000 ^">LR822067.1" \
       GCA_902655055.2_mLutLut1.2_genomic.fna \
       > GCA_902655055.2_mLutLut1.2_genomic_mt.fasta

## Loop for each cram file, converting to F and R, trimmed fq reads
INDS=($(for i in /path/to/*cram
do
        echo $(basename ${i%.*})
done))

for IND in ${INDS[@]}
do
	CRAM=/path/to/${IND}.cram
	CRAM_SORT=${IND}_sorted.cram
	
	# sort reads in cram file
	samtools sort -n -@ 32 $CRAM -o $CRAM_SORT
	# convert cram to fq
	samtools fastq -@ 32 -T $REF $CRAM_SORT -1 ${IND}_1.fq.gz -2 ${IND}_2.fq.gz
	# trim adapters
	java -jar $TRIMMOMATIC PE \
		${IND}_1.fq.gz ${IND}_2.fq.gz \
		${IND}_FP.fq.gz ${IND}_FUP.fq.gz \
		${IND}_RP.fq.gz ${IND}_RUP.fq.gz \
		-threads 32 \
		ILLUMINACLIP:/path/to/TruSeq3-PE.fa:2:30:10
done

# Function to run novoplasty, where $1 is the config input file
function run_novoplasty {
        perl NOVOPlasty-master/NOVOPlasty4.3.1.pl -c $1
}
export -f run_novoplasty

## Loop for each otter (5x cram files)
LOOP2=($(for i in /path/to/*cram
do
	echo $(basename ${i})
done))

for LOOP in ${LOOP2[@]}
do
	temp1=${LOOP%_so*}
    new=${temp1#*#}
	mkdir directory_${new}

	echo -e "\nProject
	-----------------------
	Project name          = ${new}
	Type                  = mito
	Genome Range          = 12000-22000
	K-mer                 = 33
	Max memory            = 64
	Extended log          = 0
	Save assembled reads  = no
	Seed Input            = GCA_902655055.2_mLutLut1.2_genomic_mt.fasta
	Extend seed directly  = no
	Reference sequence    =
	Variance detection    =
	Chloroplast sequence  =
	
	Dataset 1:
	-----------------------
	Read Length           = 151
	Insert size           = 300
	Platform              = illumina
	Single/Paired         = PE
	Combined reads        =
	Forward reads         = /path/to/all_${new}_FP.fq.gz
	Reverse reads         = /path/to/all_${new}_RP.fq.gz
	Store Hash            =
	
	Heteroplasmy:
	-----------------------
	MAF                   =
	HP exclude list       =
	PCR-free              =
	
	Optional:
	-----------------------
	Insert size auto      = yes
	Use Quality Scores    = no
	Output path           = directory_${new}/\n" > config_${new}.sh
	
done

# make a list of all the input config files
ls config_* > list

# run the novoplasty function in parallel over the list of config files
parallel --verbose -j 32 run_novoplasty :::: list
```

## Summary of mitobim.sh

Aim: Example to run mitobim on a single sample, for which novoplasty failed.

```
#!/bin/bash

module load samtools/1.13
module load fastp/v0.20

REF=GCA_902655055.2_mLutLut1.2_genomic.fna

grep -A 10000 ^">LR822067.1" $REF > GCA_902655055.2_mLutLut1.2_genomic_mt.fa

## Run fastp in a loop to merge reads
INDS=($(for i in /path/to/*.cram
do
        echo $(basename ${i%.*})
done))

for IND in ${INDS[@]}
do
    CRAM=/path/to/${IND}.cram
    CRAM_SORT=${IND}_sorted.cram
    # sort reads in cram file
	samtools sort -n -@ 16 $CRAM -o $CRAM_SORT
    # convert cram to fq
	samtools fastq -@ 16 -T $REF $CRAM_SORT -1 ${IND}_1.fq.gz -2 ${IND}_2.fq.gz
	# merge forward and reverse fq reads with fastp
	fastp -i ${IND}_1.fq.gz -I ${IND}_2.fq.gz -m --merged_out ${IND}_merged.fastq
done

# combine all fastq for 1 sample from the 5x cram files, into 1 sample fq file
cat *merged.fastq > mLutLut7_merged.fastq

# write configuration file
echo -e "\n#manifest file for basic mapping assembly with illumina data using MIRA 4

project = mLutLut7

job=genome,mapping,accurate

parameters = -DI:trt=/tmp/mLutLut7 -NW:mrnl=0 -AS:nop=1 SOLEXA_SETTINGS -CO:msr=no

readgroup
is_reference
data = GCA_902655055.2_mLutLut1.2_genomic_mt.fa
strain = mLutLut7

readgroup = reads
data = mLutLut7_merged.fastq
technology = solexa
strain = mLutLut7\n" > manifest_mLutLut7.conf

# Run mira
mkdir -p /tmp/mLutLut7
export LC_ALL=C
/path/to/mira manifest_mLutLut7.conf
rm -rf /tmp/mLutLut7

# Run MITObim.pl
module load perl/5.34.0
perl /path/to/MITObim.pl \
	--mirapath /path/to/mira_4.0.2/ --NFS_warn_only \
	-start 1 -end 10 -sample mLutLut7 -ref mLutLut7 -readpool mLutLut7_merged.fastq \
	-maf mLutLut7_assembly/mLutLut7_d_results/mLutLut64_out.maf &> log
```

## Summary of psmc.sh

Aim: Running PSMC in a loop over samples.

```
#!/bin/bash

module load bcftools/1.14
module load samtools/1.13
module load perl/5.34.0

REF=GCA_902655055.2_mLutLut1.2_genomic.fna
BED=onlychrom.bed

FILES=($(for i in *fa
do
        echo $(basename ${i%_mt*})
done))

for FILE in ${FILES[@]}
do
	DV_IN=mLutLut_renamed_autosomes_bisnps.vcf.gz
	CONSENSUS=${FILE}_onlychrom_consensus.fa
	PSMCFA=${FILE}_onlychrom.psmcfa
	PSMC=${FILE}_onlychrom.psmc

	# make consensus sequence
	cat $REF | bcftools consensus --haplotype I -s $FILE $DV_IN > $CONSENSUS
	gzip $CONSENSUS
	
	# transform the consensus sequence into a fasta-like format where the i-th character in the output sequence indicates whether there is at least one heterozygote in the bin (100i, 100i+100).
	/path/to/psmc-master/utils/fq2psmcfa -q20 ${CONSENSUS}.gz > $PSMCFA

	# running psmc (human settings)
	/path/to//psmc -N25 -t15 -r5 -p "4+25*2+4+6" -o $PSMC $PSMCFA

done
```

## Summary of psmc\_plotting.sh

Aim: Plotting PSMC results.

```
#!/bin/bash

module load bcftools/1.14
module load samtools/1.13
module load perl/5.34.0

FILES=($(for i in ${IN_PATH}/*.psmc
do
        echo $(basename ${i%_onl*})
done))

for FILE in ${FILES[@]}
do
	# specify mutation rate and generation time
	MUT=8.64e-09
	GEN=6

	PSMC=${IN_PATH}/${FILE}_onlychrom.psmc
	OUT=${FILE}_onlychrom_a${MUT}_g${GEN}
	
	/path/to/psmc_plot.pl $OUT $PSMC	
	perl /path/to/psmc_plot.pl -u $MUT -g $GEN -R $OUT $PSMC

done
```
