# Supplementary material for: Genomics Reveals Complex Population History and Unexpected Diversity of Eurasian Otters (Lutra lutra) in Britain Relative to Genetic Methods
Source: Mol Biol Evol. 2023 Sep 15;40(11):msad207. doi: 10.1093/molbev/msad207 (PMC10630326; doi:10.1093/molbev/msad207)
Supplement: msad207_Supplementary_Data [file msad207_supplementary_data.zip › SM2.pdf]

Genomics reveals complex population history and unexpected diversity of Eurasian otters (*Lutra lutra*) in Britain relative to genetic methods

du Plessis, Sarah J.[1+], Blaxter, Mark[2], Koepfli, Klaus-Peter[3,4], Chadwick, Elizabeth A.[1], Hailer, Frank[1+]

[1] School of Biosciences, Sir Martin Evans Building, Cardiff University, Museum Avenue, Cardiff, CF10 3AX, Wales, UK.

[2] Tree of Life, Wellcome Sanger Institute, Wellcome Genome Campus, Hinxton, Cambridge, CB10 1SA, UK.

[3] Smithsonian-Mason School of Conservation, George Mason University, Front Royal, VA 22630, USA.

[4] Centre for Species Survival, Smithsonian's National Zoo and Conservation Biology Institute, Washington DC, 20008, USA.

[+]Correspondence to: duPlessisS@cardiff.ac.uk; HailerF@cardiff.ac.uk

Supplementary Material

Table of Contents

Table of Contents 2
1 Sample collection 3
2 Data processing, depth and breadth of coverage 4
3 Microsatellite conditions 6
4 Locations of previously identified *L. lutra* microsatellite loci in the reference genome 7
5 Microsatellite summary statistics 10
6 Population structure 11
6.1 fineSTRUCTURE 13
7 Relatedness 16
8 Genetic diversity 17
9 Demographic analyses 19
10 National survey data 25
11 Mitochondrial genome analyses 26
12 Runs of Homozygosity methods 32
13 References 33

1 Sample collection

The Cardiff University Otter Project (CUOP) has archived samples from >4,000 Eurasian otters across England, Scotland and Wales, dating back to 1992. Samples are primarily from individuals found dead, typically due to road traffic accidents. Once collected, carcasses were stored and transported frozen. Carcasses were then stored at room temperature for 24 hours before muscle tissue was sampled from the hind right leg and dry frozen. Informed by a prior genetic assessment of the species in Britain based on 15 microsatellite loci (Stanton et al. 2014), 45 individuals for the present study were selected based on location found, mapped using ArcGIS Pro 2.7.0, aiming to represent the genetically distinct stronghold populations, while avoiding known admixture zones. These strongholds were (1) the North of England and Scotland, (2) Southwest England, (3) Wales, and (4) central and eastern England.

2 Data processing, depth and breadth of coverage

Specimens for resequencing were derived from four previously defined source populations, (1) the North of England and Scotland, (2) Southwest England, (3) Wales, and (4) central and eastern England. Whole genomes of 45 otters across the four source populations were sequenced using Illumina NovaSeq 150 bp paired-end reads to a depth of coverage ranging from 16 to 40-fold, and breadth of coverage from 80 to 98% (Figure 1, SM1).

The core Sanger informatics team, specifically the New Pipeline Group (NPG), quality checked and aligned this data using their standard alignment pipeline. Briefly, Illumina BCL was first converted to cram format, demultiplexing by sample, using samtools v1.10 (Danecek et al. 2021) and biobambam2 v2.0.79 (https://gitlab.com/german.tischler/biobambam2). Second, the unaligned sample-level cram files were merged across lanes, aligned to the mLutLut1.2 reference genome (Mead et al. 2020), phiX spike reads removed, duplicates marked, and output to final aligned product cram files. These stages used bwa v0.7.17 (Li and Durbin 2009), scramble v1.14.9 (https://github.com/jkbonfield/io_lib) and bambi v0.13.1 (https://github.com/wtsi-npg/bambi).

Depth and breadth of coverage were calculated from the DToL cram files. Files were converted from cram to bam format, using the reference genome (mLutLut1.2) and samtools v1.10 view and index. Each sample was combined from 5 cram files to create 45 sample-level bam files, and indexed using samtools merge. Visual quality check, number of raw reads and raw read GC% were assessed using fastQC (Andrews 2010). Samtools flagstat was used to check the number of reads mapped, and to find depth and breadth of coverage, samtools coverage was used for each sample bam file (SM1).

To call variants, cram files for each sample were merged into a single bam file per sample using samtools v1.9 'merge'. DeepVariant v1.1.0 (Poplin et al. 2018; Yun et al. 2020) was used to call variants on each bam file separately using the following command 'run_deepvariant --

model_type=WGS –ref GCA_902655055.2_mLutLut1.2_genomic.fna --reads bam/$1.bam --output_vcf gvcf/$1.vcf.gz --output_gvcf gvcf/$1.gvcf.gz’. GLNexus v1.2.2 (Yun et al. 2020) was used to merge individual gVCFs using the command ‘glnexus_cli --config DeepVariantWGS gvcf/*.gvcf.gz > all.bcf.gz’. As the DeepVariant and GLNexus machine learning algorithms used to call variants contain strict internal filtering, no further filtering was conducted on this dataset unless otherwise stated. From this vcf, we identified 8,931,760 biallelic SNPs from chromosomes 1 to 18.

3 Microsatellite genotyping

The microsatellite PCR protocol was used from Hobbs *et al.* (2006). Primer multiplexes were made of equal proportion from each primer pair. For example, 2 µl of Multiplex 1 primers was made from 0.2 µl of each forward and reverse primer for each of the 5 primer pairs in that multiplex (see SM1 for more primer specific details). Qiagen Multiplex PCR Kits were used to conduct PCRs in a total volume of 10 µl, using 5 µl Master Mix, 2 µl primers, 1 µl water and 2 µl DNA extract per sample reaction. Negative and positive controls were used throughout DNA extraction and PCR processing to ensure contamination did not occur. The PCR for all multiplexes consisted of 15 minutes at 95 °C, followed by 29 cycles of 30 seconds at 94 °C, 90 seconds at 58 °C and 60 seconds at 72 °C, before a final stage of 30 minutes at 60 °C and samples being held at 14 °C. Samples were diluted 1:10 before being sent for fragment analysis (see main text).

The expected range of repeat lengths were updated (SM1) based on these results, leading to two loci from multiplex 1, using the same dye, overlapping in four samples (Lut435 ranging 115-143 bp, and 04OT22 ranging from 136-164 bp). After running example samples using single loci primers and comparison to multiplex results, all four samples could be scored due to the distinction between peaks from each locus. The lower boundary of the expected range of Lut615 was also decreased, due to the presence of a single allele significantly lower than the prior range (from 214 to 195 bp) identified following replicated PCR runs. Samples which showed alleles overlapping between Lut435 and 04OT22, and samples with the lower allele in Lut615 were all from samples from the East population.

4 Locations of previously identified *L. lutra* microsatellite loci in the reference genome

As commonly used tools in applied and conservation genetics, knowledge of the locations of microsatellite loci in the genome is important for consideration of aspects such as linkage. A total of 25 microsatellite loci for Eurasian otters were identified on NCBI in a search on 22/04/2022. These loci include the 15 microsatellite markers genotyped as part of this study. To locate the loci in the Eurasian otter genome, BLAST v2.12.0 (Altschul et al. 1990) was used to make a database from the reference genome (mLutLut1.2, (Mead et al. 2020)), and the fasta sequences of the microsatellite loci were matched against this database.

All 25 sequences produced at least one hit, but two loci had significantly more (Lut604 and Lut615 had 364 and 4,876 results respectively, Table 1, Figure 1), suggesting a potential lower specificity for these markers. All results were assessed using available metrics (% identical matches, alignment length, number of mismatches, expect value and bit score) and assigned to a genomic region where possible. Microsatellites with multiple hits all had one result which was a significantly better fit based on the available metrics (Table 1). Of the 18 autosomal chromosomes, 12 contained at least 1 microsatellite loci. The smallest distance between any two loci was on chromosome 15 (LR738417) at 1.34 Mbp apart (Lut435 and 04OT17), closely followed by the remaining two loci on this chromosome with the distance between all four being 10.5 Mbp. One locus, 04OT19, was mapped to the X chromosome (LR738421).

We note that these generally large inter-locus distances for our 15 genotyped microsatellite loci are consistent with the absence of significant linkage disequilibrium signals in our data and in previous studies (see main text).

Table 1. Number of hits for each microsatellite sequence within the Eurasian otter reference genome, located using BLAST, followed by the assessment metrics for the best fitting hit for each microsatellite.

Microsatellite	Number of hits	Chromosome	% Identical matches	Alignment length	Number mismatches	Expect value	Bit score
04OT02	2	LR738408.1	99.007	302	0	7.21E-151	538
04OT04	2	LR738407.1	96.203	237	5	7.63E-105	385
04OT05	1	LR738405.1	96.866	351	2	3.56E-163	579
04OT07	1	LR738412.1	98.634	366	2	0	645
04OT14	1	LR738417.1	93.627	204	1	7.54E-78	294
04OT17	1	LR738417.1	95.88	534	1	0	845
04OT19	1	LR738421.1	97.409	386	0	0	649
04OT22	11	LR738411.1	97.175	177	0	6.85E-78	294
Lut435	1	LR738417.1	94.649	598	9	0	918
Lut453	1	LR738419.1	97.692	520	9	0	907
Lut457	4	LR738406.1	98.397	312	3	2.61E-155	553
Lut604	364	LR738403.1	97.445	548	2	0	926
Lut615	4876	LR738418.1	97.266	512	4	0	863
Lut701	1	LR738403.1	99.634	546	2	0	1000
Lut715	2	LR738419.1	100	360	0	0	665
Lut717	1	LR738417.1	96.838	253	0	2.11E-114	416
Lut733	1	LR738407.1	97.345	339	1	2.11E-160	569
Lut782	1	LR738408.1	98.653	297	1	1.44E-146	523
Lut801	2	LR738420.1	100	359	0	0	664
Lut818	2	LR738410.1	98.623	363	1	0	640
Lut832	1	LR738407.1	98.765	324	0	1.53E-161	573
Lut833	1	LR738412.1	93.415	410	3	1.99E-166	590
Mel09	1	LR738420.1	88.889	90	7	3.18E-23	111
Mel10	1	LR738403.1	92.045	88	0	6.73E-25	117
Mel14	1	LR738415.1	95.27	148	0	5.39E-58	228

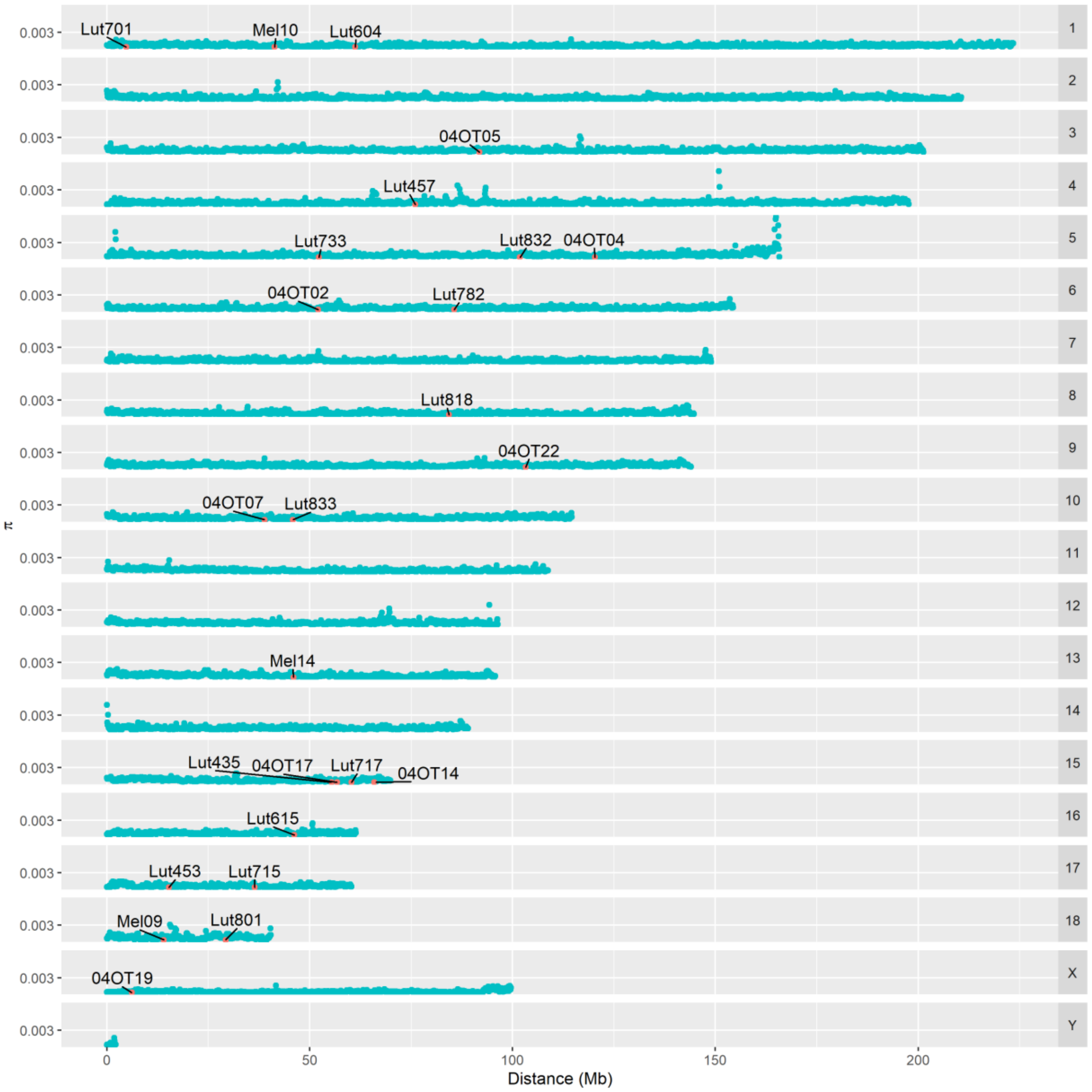

Figure 1. Nucleotide diversity (π) along *L. lutra* reference genome chromosome-level scaffolds, calculated for 100 kbp windows across all 45 UK otter samples. Inferred microsatellite loci positions, based on the best fitting BLAST hits, are shown by red dots and labelled.

5 Microsatellite summary statistics

Table 2. Genetic diversity statistics calculated based on microsatellite data by population of Eurasian otters in Britain. n, number of individuals, H_O observed heterozygosity, H_E expected heterozygosity, A number of alleles, A_R allelic richness, F_{IS} inbreeding coefficient, presented as means across loci, with standard error in brackets.

Population	n	H_O	H_E	A	A_R	F_{IS}
East	12	0.63 (0.04)	0.70 (0.03)	5.20 (0.31)	4.53 (0.26)	0.15
North	13	0.64 (0.03)	0.70 (0.02)	5.13 (0.43)	4.37 (0.31)	0.13
Southwest England	8	0.48 (0.06)	0.49 (0.05)	3.20 (0.30)	2.97 (0.25)	0.08
Wales	12	0.53 (0.04)	0.53 (0.03)	3.60 (0.16)	3.26 (0.13)	0.03

Table 3. Private alleles of microsatellite loci by population of Eurasian otters in Britain. Of the 15 microsatellite loci included in this study, the 12 loci below identified alleles private to one or more of the populations.

	East	North	Southwest England	Wales
Lut435	1	4	0	0
Lut453	0	1	1	0
Lut717	1	1	0	0
04OT22	1	0	0	0
Lut604	1	0	0	0
Lut733	1	0	0	0
Lut615	0	1	0	0
Lut902	2	0	0	0
Lut782	0	2	0	0
Lut701	2	1	0	0
Lut833	0	1	0	0
Lut715	1	0	0	0
Total	10	11	1	0

6 Population structure

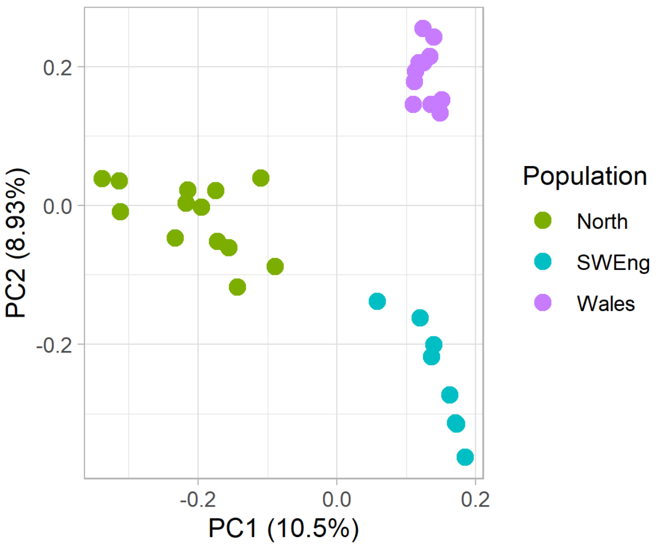

Figure 2. Principal component analysis of whole genome SNPs from Eurasian otter samples from populations in the North, Southwest England, and Wales (excluding samples from the East population).

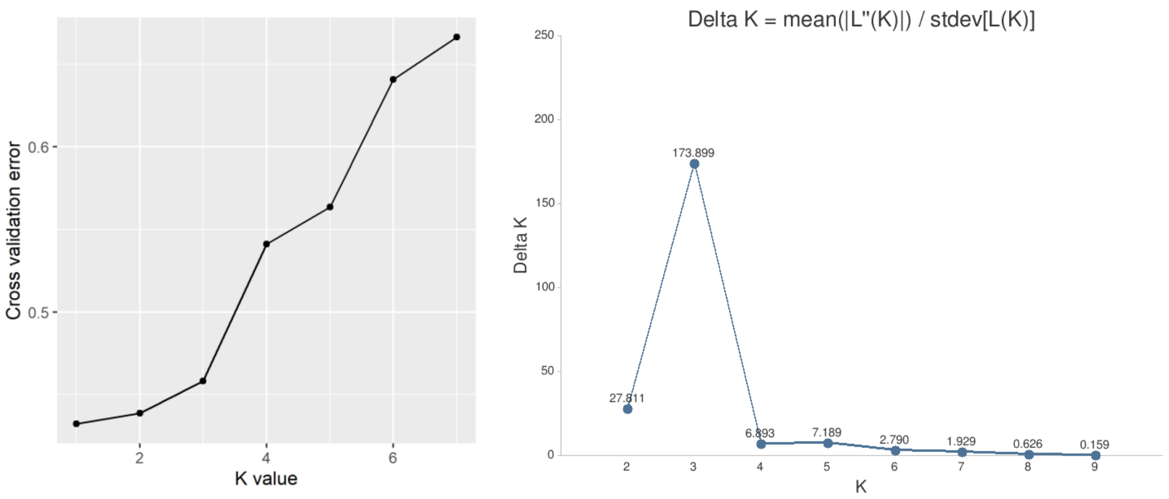

Figure 3. Cross-validation error for ADMIXTURE K values 1 to 7 (genomic SNPs), and Δ K for STRUCTURE K values 1 to 10 (microsatellite data).

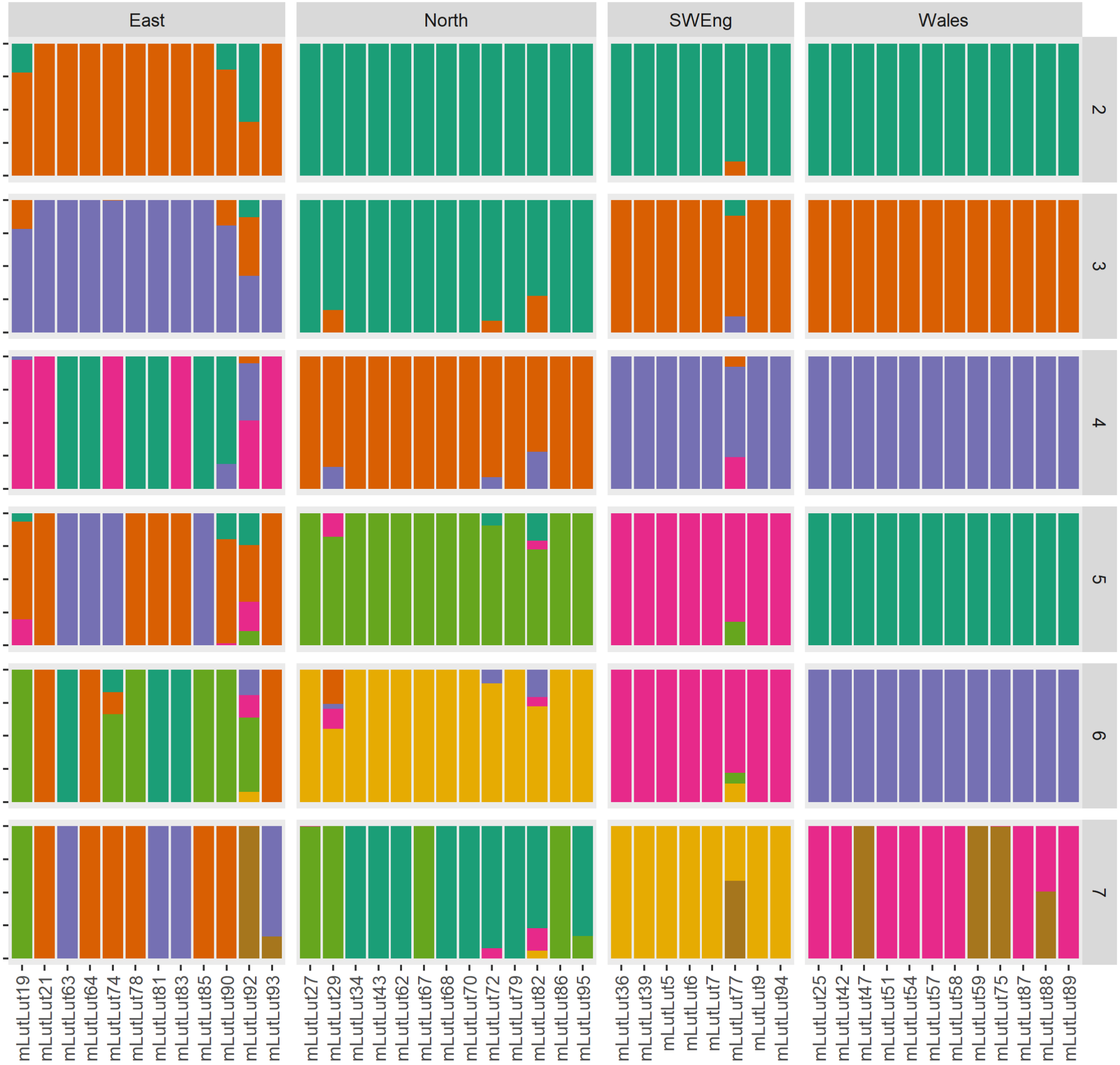

Figure 4. Population structuring of British Eurasian otters as assessed by running ADMIXTURE on whole genome SNPs, with K values from 2 to 7 (could not converge at K=8 and above).

6.1 fineSTRUCTURE

To run fineSTRUCTURE v4.1.1 (Lawson et al. 2012), missing data were removed, and samples were phased using SHAPEIT4 (Delaneau et al. 2019). The data were reformatted using the provided impute2chromopainter.pl script for each chromosome-level scaffold separately. Recombination rate maps were produced using the provided makeuniformrecfile.pl script, assuming a constant rate of recombination per base. fineSTRUCTURE was run in the automatic mode, using the default settings, and results were plotted using the provided R functions scripts.

Obtained signals of population structuring were consistent between linked (using recombination rates; Figure 5) and unlinked (without recombination rates; Figure 6) runs of fineSTRUCTURE, although with clearer differentiation between non-eastern populations in the unlinked run.

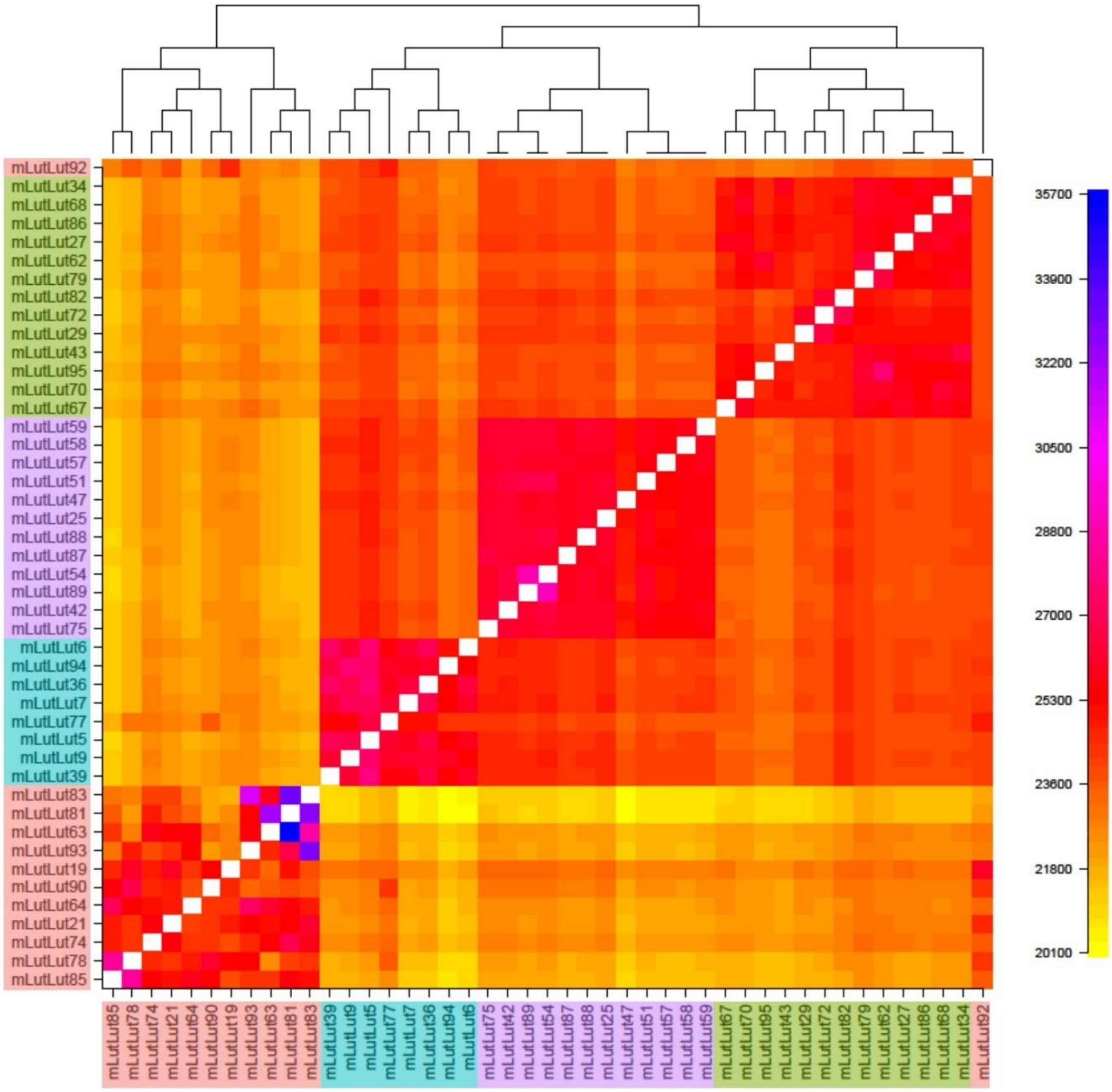

Figure 5. fineSTRUCTURE coancestry matrix presented as a heatmap where cell colour represents estimated shared genetic ancestry from high (blue) to low (yellow), run on whole genome SNP data for British Eurasian otter samples. Sample IDs colour coded by population origin, East (red), Southwest England (blue), Wales (purple) and North (green).

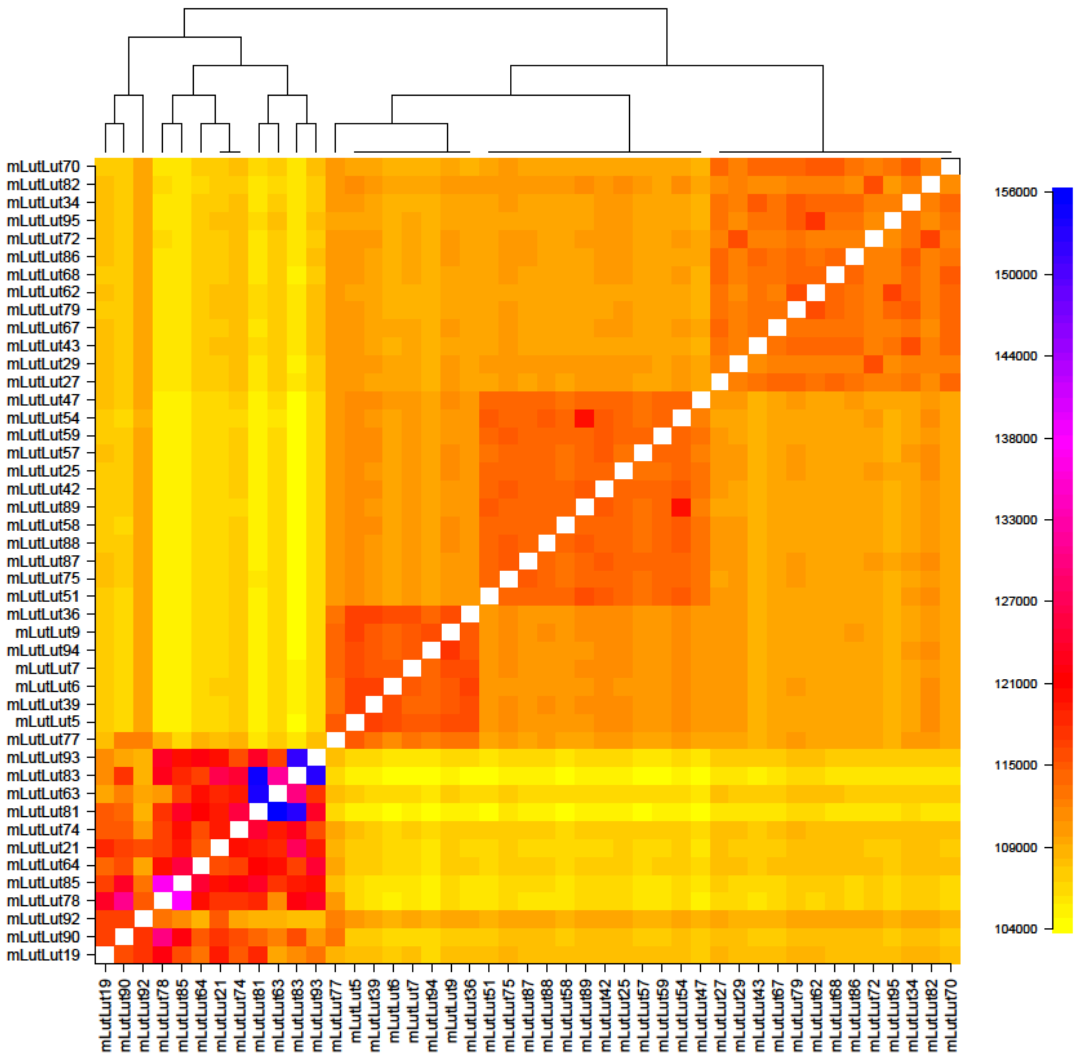

Figure 6. fineSTRUCTURE coancestry matrix presented as a heatmap where cell colour represents shared coancestry from high (blue) to low (yellow), among Eurasian otter samples within the UK, run as an unlinked model i.e. not accounting for linkage or recombination among loci.

7 Relatedness

VCFtools was used to calculate pairwise relatedness among samples using the method of Manichaikul et al. (2010).

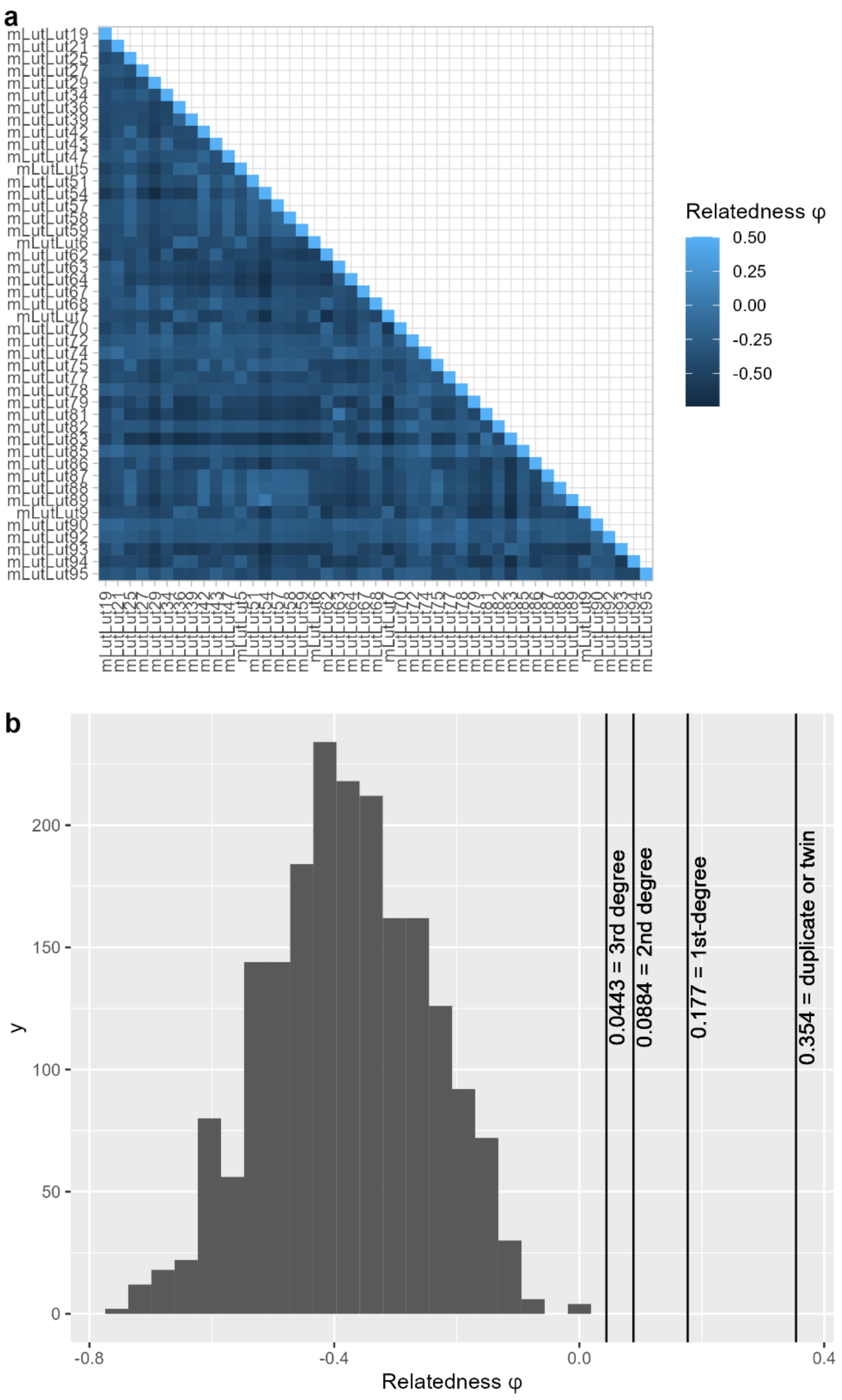

Figure 7. Histogram of relatedness calculated among sample pairs (a) as a pairwise heatmap, and (b) histogram, with vertical lines indicating expected cut-off values for each degree of relatedness (1st to 3rd degree and twins). This suggests no samples in our study show a 3rd degree of relatedness or closer.

8 Genetic diversity

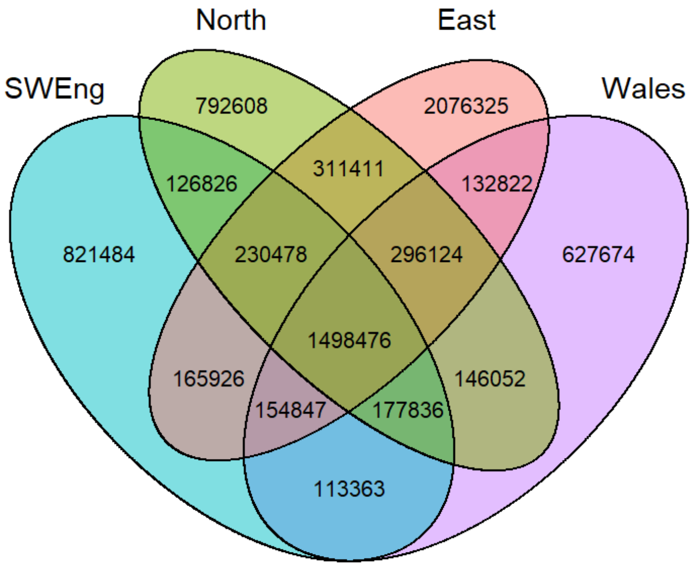

Figure 8. Private SNP counts measured across a random subsample of 8 individuals from each population.

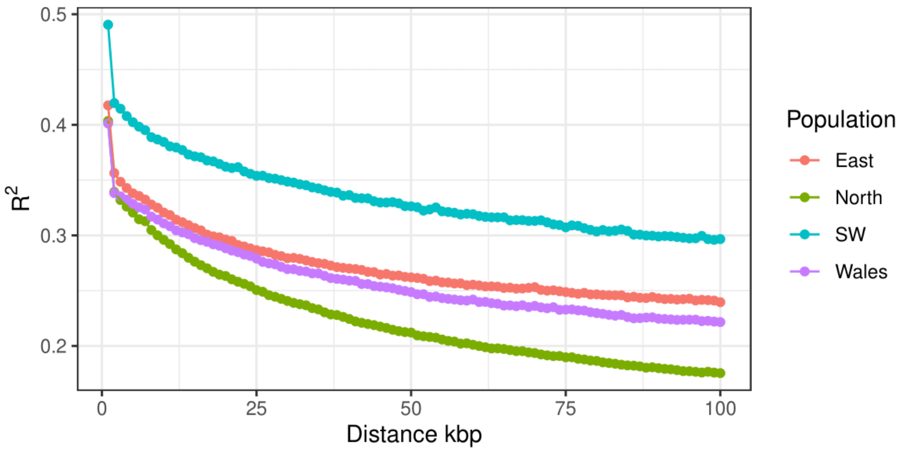

Figure 9. Linkage disequilibrium between pairs of SNPs against distance between them, up to 100 kbp apart on chromosome 1 (LR738403), calculated for each population of Eurasian otters in Britain.

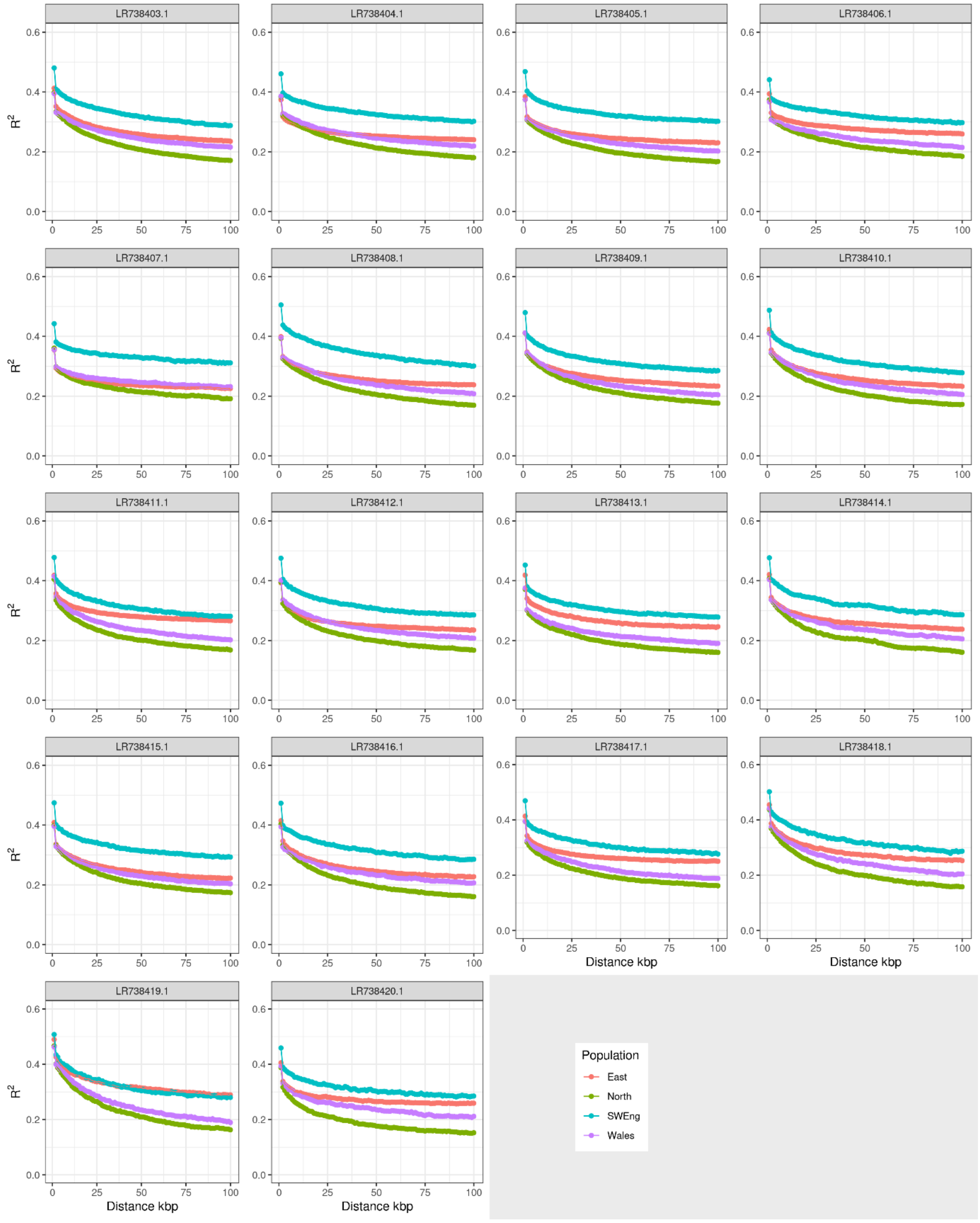

Figure 10. Linkage disequilibrium between pairs of SNPs against distance between them, up to 100 kbp apart, calculated within each population and for each chromosome separately.

9 Demographic analyses

We used GONE (Santiago et al. 2020) to estimate recent effective population size (N_e). GONE calculates LD between pairs of SNPs over a range of recombination rates and finds the series of N_e that best explains the observed LD spectrum (Santiago et al. 2020). Samples were grouped into populations based on their geographic origin and GONE was run on each population subset using the default settings of no MAF pruning, removing ungenotyped SNPs, maximum recombination rates of 0.05, and 40 internal replications. Phasing was not used, and because recombination rate for the species is not known, the suggested 1 cM/Mb was used (Wong et al. 2010; Santiago et al. 2020). N_e estimates were improbably high when GONE was run for 200 generations, and significantly lower when run for 2,000 generations (as is the default). Therefore, we ran all simulations for 2,000 generations, but only present results for the most recent 200 generations, as this is when the model is most effective at detecting changes in N_e (Santiago et al. 2020). N_e over 2,000 generations was calculated in 400 bins, therefore resulting in an estimate every 5 generations. We repeated each run 10 times, with each run taking a new subsample of SNPs (comparable to bootstrapping), to assess variance in N_e estimates across runs. We found that using a larger subset of SNPs (100,000) per autosomal chromosome, than the default (50,000) resulted in smaller variance between bootstrap runs, and therefore used 100,000 SNPs for all runs. We predicted that the British bottleneck occurred in the 1950-80s, corresponding to 9 to 17 generations before these samples were collected (2016-2020), based on a generation time of 4 years (see below).

Pairwise sequentially Markovian coalescent (PSMC) inference (Li and Durbin 2011) was used to estimate older effective population size (N_e) changes. Variants identified from the DeepVariant-called vcf file were applied to the reference genome to produce a consensus sequence for each sample (using bcftools 'consensus' command). These sequences were then converted to psmcfa format, and PSMC was run using the example settings, suitable for humans (-N25 -t15 -r5 -p "4+25*2+4+6"), based on the PSMC GitHub page (https://github.com/lh3/psmc). Results were plotted using the mutation rate of 8.64e-9 per site, per generation, as estimated by Beichman *et al.*

(2019) for sea otters (*Enhydra lutris*) based on the divergence time and genomic differentiation between sea otters and ferrets (*Mustela putorius furo*, Figure 11). Estimates of N_e from less than 10,000 years ago were not plotted, due to potential limitations of the coalescent method in inferring population size more recently than this.

Both previous methods require estimates of generation time, and in Eurasian otters this varies from 3 to 5 years, depending on the population assessed and method used (Heggberget 1988; Pertoldi et al. 2001; Arrendal et al. 2007; Tison et al. 2015; de Ferran et al. 2022). Reproductively active female otters in Germany were found to be mainly 6-9 years in age, whereas in a comparative British study only 2 in 110 females were found to be aged 6 or over (Hauer et al. 2002; Sherrard-Smith and Chadwick 2010). However, both studies were conducted on road traffic accident animals and therefore potentially biased towards younger, migrating individuals. Due to the uncertainty around generation time, 4 years is presented in the results for both GONE and PSMC, with results from 2, 4 and 6 years presented below (Figure 12 and Figure 13). Bootstrapping was performed on a subset of 7 samples for PSMC (Figure 14).

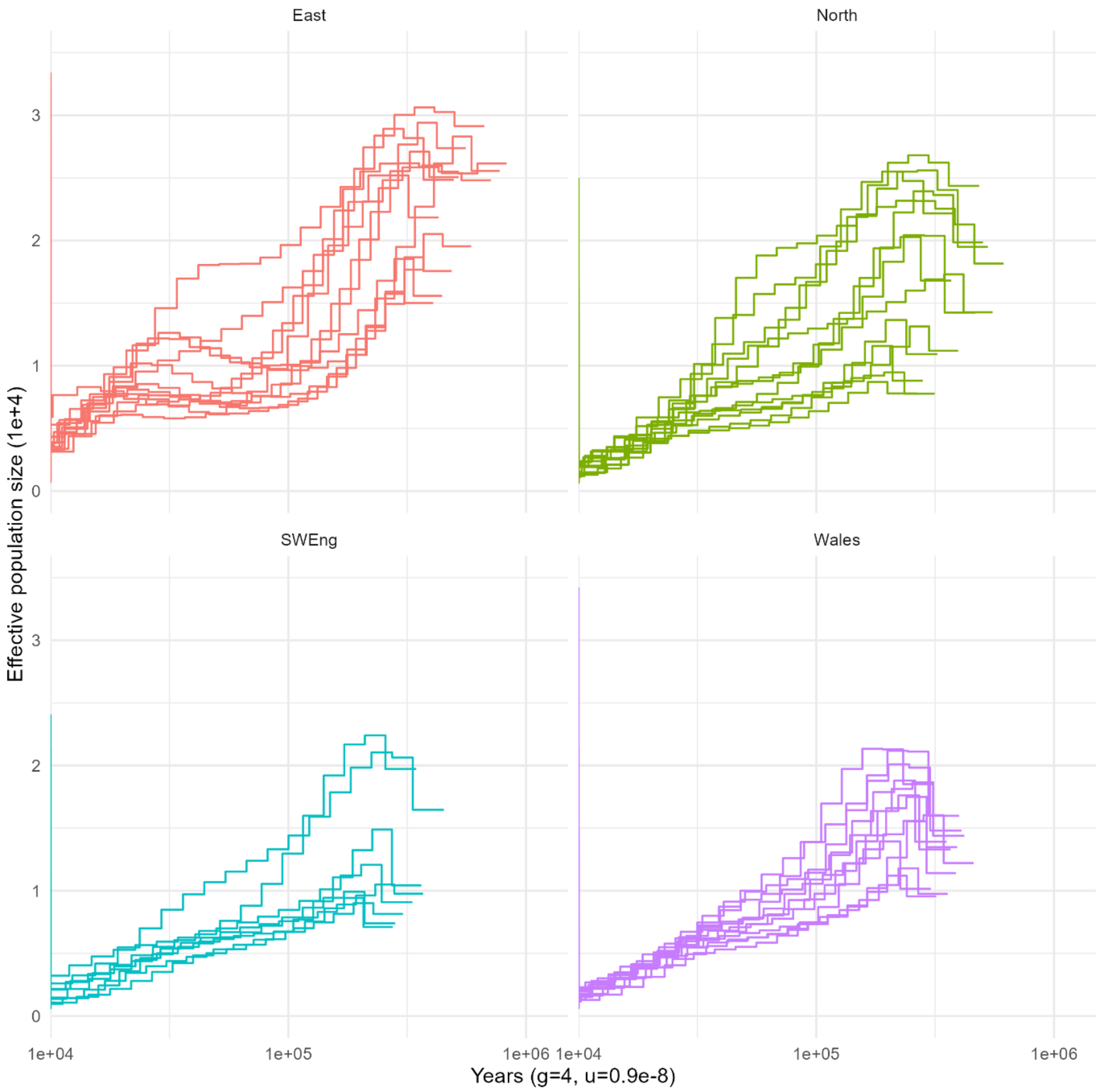

Figure 11. Effective population size across time as calculated by PSMC. Calculated on an individual level basis on samples sizes of n=8 for Southwest England, n=12 for Wales and East, and n=13 for North, assuming a generation time of 4 years and mutation rate of 8.64e-9 per site, per generation.

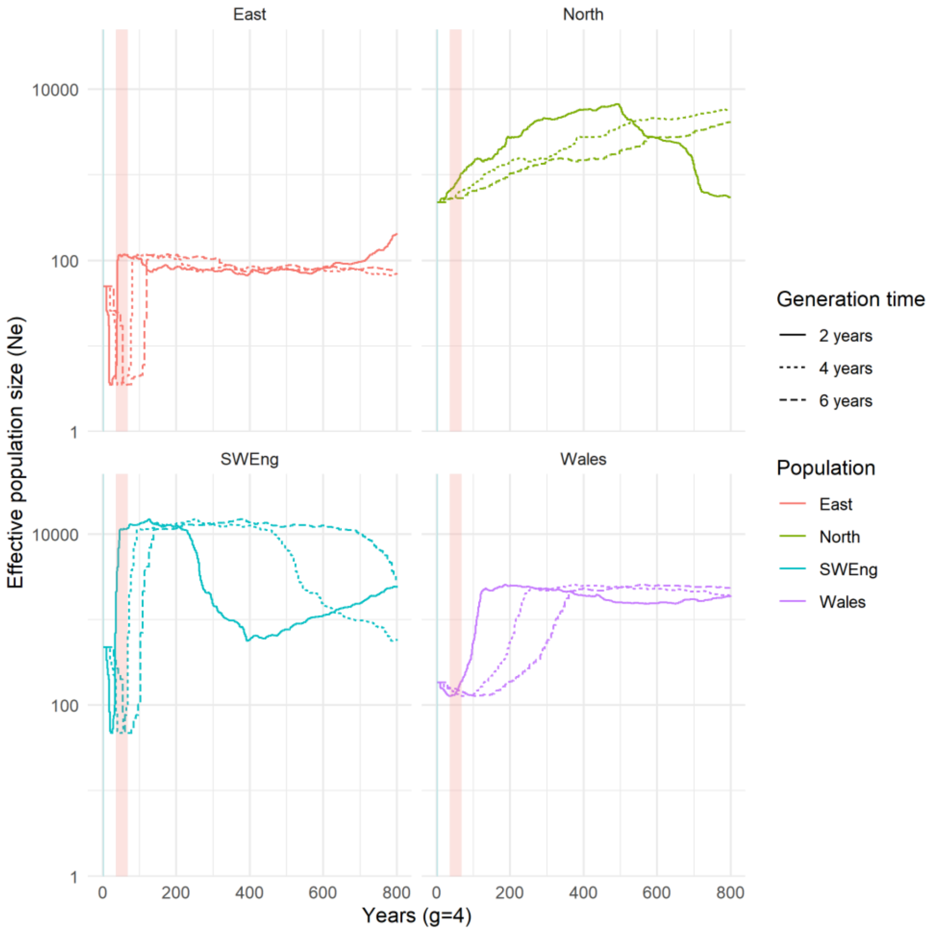

Figure 12. Effective population size over time as calculated by GONE for 1 bootstrap run for each population, plotted using a generation time of 2, 4 and 6 years in solid, short dash and long dashed lines, respectively.

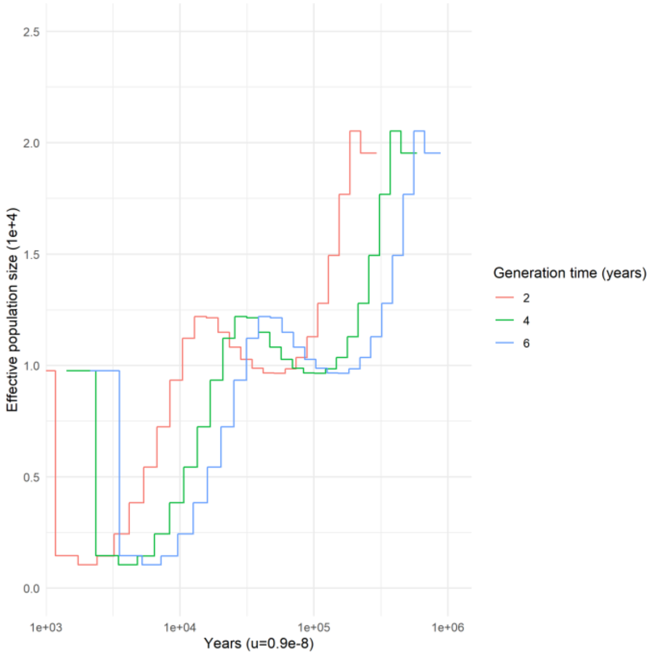

Figure 13. Effective population size across time as calculated by PSMC for sample mLutLut21 (East), using an estimated mutation rate of 0.9e-8 per site, per generation, and generation times of 2, 4 and 6 years in red, green and blue, respectively.

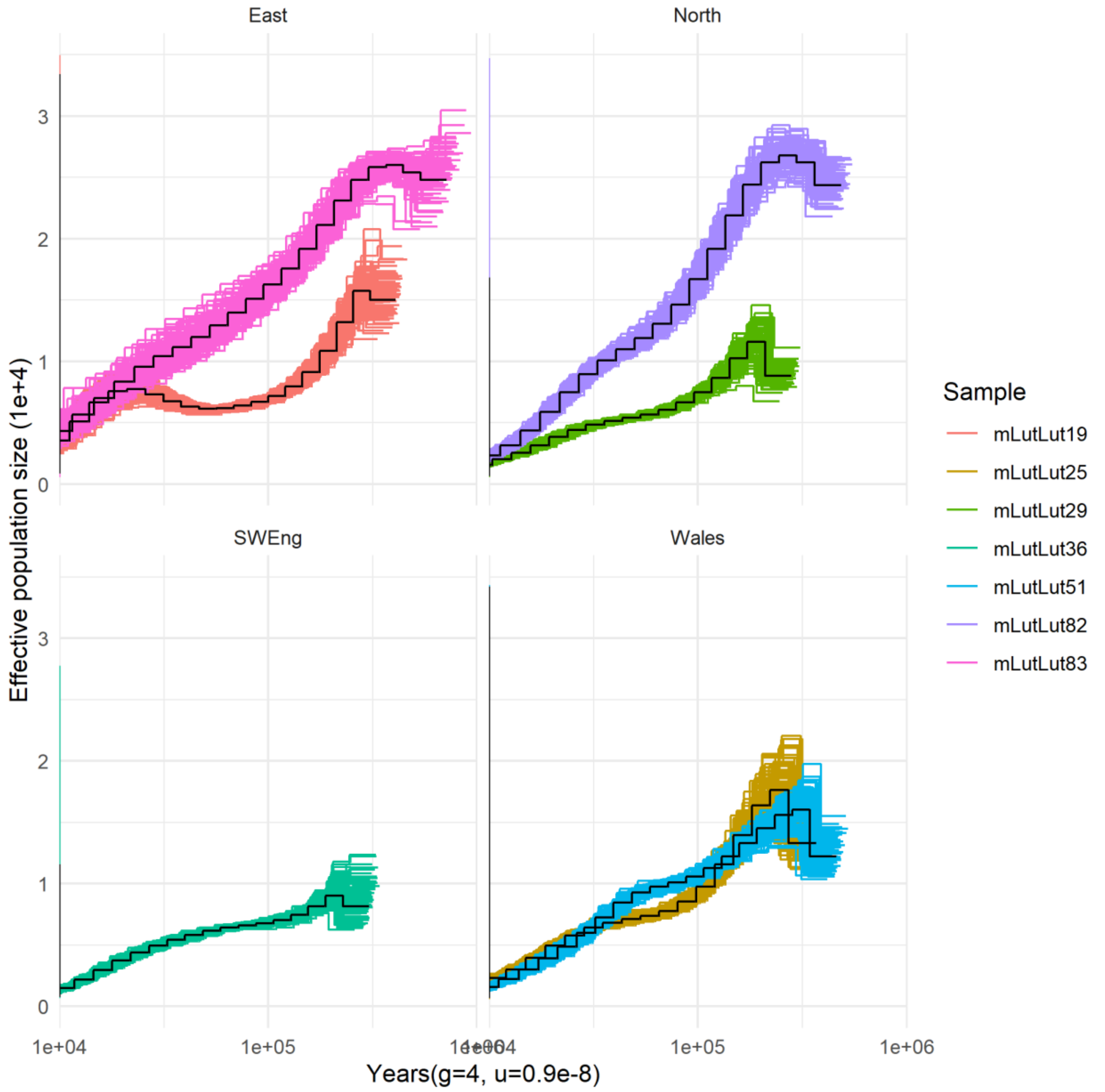

Figure 14. Effective population size across time as calculated by PSMC for a subset of seven samples, using an estimated mutation rate of 0.9e-8 per site, per generation, and generation times of 4, with 100 bootstrap replicates per sample.

10 National survey data

Recent estimates of Ne using GONE were compared to survey data from five National Otter Surveys of England and Wales and four surveys of Scotland, from 1977 to 2010 (Strachan et al. 1990; Crawford and Scholey 2010; Kean and Chadwick 2021). Data were only included for sites that were surveyed in all surveys, therefore leading us to exclude large regions of Scotland (primarily the highlands and islands) which were not surveyed in 1984-85. This resulted in the proportion of positive sites for our subset to be a slight underestimate relative to all of Scotland as the proportion of positive sites in the regions excluded were high. For the Welsh surveys, the number of positive sites was calculated from the percentages and number of surveyed sites. Surveys were conducted on river basin scale, and therefore assigned to stronghold regions for this study as given in SM1, with the exception of Trent, which lies centrally between strongholds and therefore was excluded. Regions assigned to the Northern stronghold showed disparity among trends between those surveyed in the English and Scottish surveys. It is possible that these differences arose due to methodological differences, but more likely that this reflects the differences between the extent of the bottleneck between the regions of northern England and Scotland. Rather than calculating proportion of positive sites across these regions, they have been plotted separately and therefore do not align with the strongholds discussed throughout the remainder of this study.

11 Mitochondrial genome analyses

The Eurasian otter reference mitochondrial genome sequence was assembled using both long and short reads using mitoVGP (Formenti et al. 2021), circumventing challenges that could arise from *de novo* assembly of mitochondrial genomes from short reads alone. For Eurasian otters, (Formenti et al. 2021)a tandem repeat region was identified within the control region/D-loop, but there was no evidence of any gene duplications (Formenti et al. 2021). Because in the present study we relied on short read sequencing data alone, we remained unable to resolve the tandem repeat region. Hence, we acknowledge that we are producing near-complete mitochondrial genome sequences, but will hereafter refer to these as whole mitochondrial genome sequences.

We assembled the mitochondrial genomes for each sample independently and combined these with previously published data and sequences. Raw fastq files were trimmed for adapter sequences and not quality, using Trimmomatic v0.39 (Bolger et al. 2014) and standard ILLUMINACLIP settings (2:30:10). NOVOPlasty v4.3.1 (Dierckxsens et al. 2017) was used to assemble the mitochondrial genomes from the adapter-trimmed reads using the 16,536 bp reference genome mitochondrial scaffold (LR822067.1) as the seed. Perl v5.34.0 was used to execute the NOVOPlasty script. Settings from the example configuration file were used, adjusting only memory allocation, seed, and input and output paths. The configuration file details the input data (such as read length, insert size and platform), an expected assembled mitochondrial genome size between 12000-22000bp, and K-mer length of 33 (indicating the allowed length of overlap between matching reads).

NOVOPlasty successfully assembled 41 samples to circular sequences but failed to assemble 4 samples with low depth and breadth of coverage (SM1). MITObim v1.9.1 (Hahn et al. 2013) is an approach to *de novo* mitochondrial genome assembly utilising MIRA v4.0.2 (Chevreux et al. 1999) iteratively, using the output of the previous iteration as the seed for the next iteration. Due to the effectiveness of this iteration-based approach, MITObim was used for the 4 samples that failed to

assemble using NOVOPlasty. Reads were sorted and converted from cram to fastq format using samtools, and forward and reverse reads were merged using fastp v0.20 (Chen et al. 2018). Default settings were used for the configuration file, such as the number of iterations specified as between 1 and 10. MITObim reached a stationary read number (indicating convergence) after 2 or 3 iterations for 3 samples but failed to assemble the remaining sample (mLutLut7).

Sequences were linearised to match the reference mitochondrial genome, and Geneious Prime v2022.0.2 (https://www.geneious.com) was used to align sequences using the MUSCLE algorithm (Edgar 2004). Initially, all assembled British sequences were aligned, and due to missing data and uncertain repeat numbers surrounding the tandem repeat, this region of the control region was removed (positions 16,050-16,202 on the reference scaffold). There was also an overhang at the end of the reference sequence (position 16,536 on the reference scaffold) in the three MITObim assembled sequences which were trimmed, leaving a total alignment length of 16,365 bp. To contextualise our samples with prior studies, 255 bp of the control region (CR) were extracted and compared to previously published control region haplotypes. These include haplotypes Lut1, Lut3 and Lut6 (Stanton et al. 2009), Lut2, Lut4 and Lut5 (Cassens et al. 2000), Lut7 (Pountney 2008), and Lut8, Lut9, Lut10, Lut11 and Lut12 (Finnegan and Neill 2010). PopArt v1.7 (Leigh and Bryant 2015) was used to produce statistical parsimony networks based on the TCS algorithm (Clement et al. 2002), and to identify the number of mutation steps between haplotypes.

We next aligned our data to previously published *L. lutra* and outgroup whole mitochondrial genome sequences. The repeat region was again removed (positions 16,035-16,289 bp relative to the reference scaffold), leaving a total alignment length of 16,392 bp. The R packages pegas (Paradis 2010) and ape (Paradis and Schliep 2019) were used to calculate summary statistics (haplotype richness, haplotype diversity and nucleotide diversity, π). IQ-TREE (Nguyen et al. 2015) ModelFinder (Kalyaanamoorthy et al. 2017) was used to identify the best fitting model based on the Bayesian Information Criterion (BIC), which was the three-substitution types model with unequal, empirical

base frequencies (Kimura 1981), and allowing for a proportion of invariable sites ('K3Pu+F+I'). A consensus tree was constructed based on 1000 bootstrap replicates (Hoang et al. 2018), and visualised using FigTree v1.4.4 ().

11.1 Presence and potential influence of NUMTs

We used NumtFinder (Edwards 2021), to search for NUMTs in the Eurasian otter reference genome, blasting the identified mitochondrial scaffold from Mead et al. (2020) against the rest of the reference genome. Due to the two divergent mitochondrial lineages identified in Britain in this study, we also searched for NUMTs using an assembled mitochondrial genome from the more frequent, alternative lineage (Lineage 3) to the reference mitochondrial scaffold (Lineage 1). This identified a range of reasonably long (from 5 to 9 kbp) and short putative NUMTs across chromosomal and unplaced scaffolds. This shows that NUMTs are indeed present in the (nuclear) reference genome of *L. lutra*.

However, upon further testing we believe we have strong evidence to suggest that the inferred mitochondrial genome lineages constitute true mitochondrial sequences and not NUMTs, for four reasons. First, the reference genome mitochondrial sequence is of the divergent Lineage 1 and was assembled from long and short reads using MitoHiFi (M. Blaxter, pers. comm.). This procedure of mitochondrial genome assembly is designed to exclude NUMTs, by identifying mitochondrial-nuclear genome boundaries, and requiring multiple, overlapping reads across this boundary to assemble it. Second, nucleotide variants differentiating the two lineages (1 and 3) span the entire mitochondrial genome, from base 84 to 16,402 (as shown in the image below), and we found no singular NUMTs which span this entire length. NumtFinder identified older and short NUMTs, in this reference genome, which have accumulated a significant proportion of mutations relative to the current mitochondrial sequence. Although these shorter NUMTs, when combined, span the entire mitochondrial genome, due to the accumulation of mutations, we found that they would not produce the inferred mitochondrial genome sequences for either lineage. Third, the vast

majority of reads used in the assembly pipeline will be true mitochondrial reads, due to the far greater proportion of mitochondrial DNA relative to nuclear DNA existing in the data. To investigate this, we mapped all reads onto to the mitochondrial genome for one sample (from mtDNA lineage 1). At the first site separating the two lineages, 16,755 reads indicated C as a base, and 120, 66, and 9 indicated A, T, G bases, respectively. Another site separating the two lineages had 17,450 reads indicating a C, and 92, 42 and 3 reads indicating bases A, G and T, respectively. When averaged across 10 randomly selected sites segregating the two divergent lineages, less than 1% of reads mapping to the site did not contain the base identified through our mitochondrial genome assembly using MITObim/Novoplasty. Although this highlights the potential presence of NUMTs found in the reference genome, or only in the sample (alongside sequencing errors), it is highly unlikely that MITObim/Novoplasty would call either site as anything other than true mitochondrial base, due to the overwhelming signal for the primary base call. Fourth, the inferred mitochondrial genome sequences show no evidence of nuclear origin in terms of substitutions unexpected for a true mitochondrial origin, for example we observe no premature stop codons or indels of any kind, in protein-coding regions.

We therefore conclude, based on the evidence we have provided, that the mitochondrial lineages identified in this study are true mitochondrial lineages and are not a reflection of NUMTs, or NUMT-derived errors.

Table 4. Summary statistics of whole mitochondrial genome diversity of Eurasian otters calculated across geographic populations within Britain. Sample size (n), number of haplotypes (nh), segregating sites (s), haplotype diversity (h) and nucleotide diversity (π).

	n	nh	s	h	π
Samples grouped by geographic population:					
East	12	5	130	0.80	0.0035
North	13	8	24	0.86	0.0005
Southwest England	7	2	2	0.48	0.0000
Wales	12	3	12	0.53	0.0003
All British samples:	44	18	153	0.93	0.0017
Samples grouped by mitochondrial lineage:					
Lineage 1	12	9	141	0.91	0.0021
Lineage 2	4	4	105	1.00	0.0024
Lineage 3	43	20	64	0.93	0.0006
L. l. nippon	1	1	-	-	-
All samples:	60	34	772	0.96	0.0041

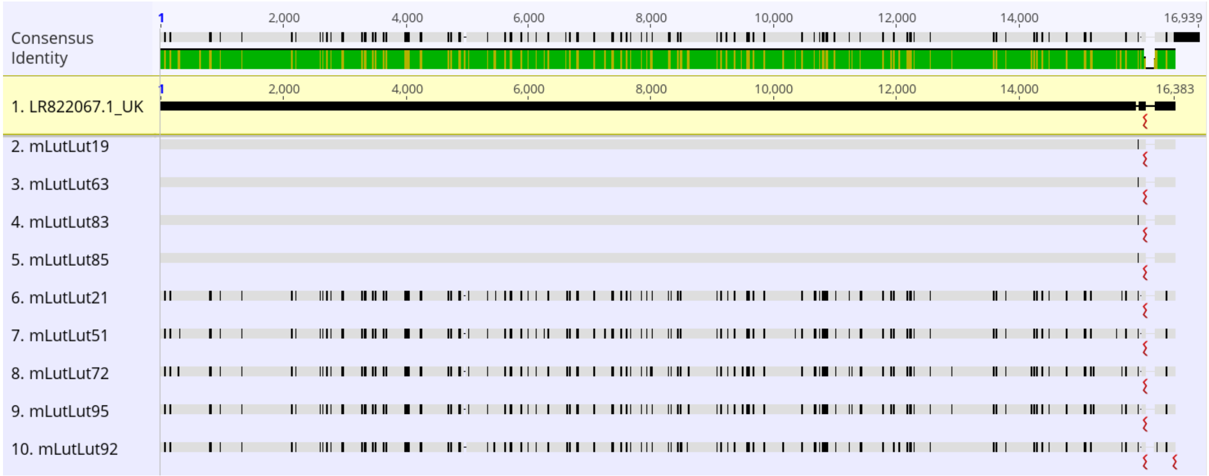

Figure 15. Visualisation of variant sites separating lineages 1 and 3 across the whole mitochondrial genome, in a subset of British Eurasian otters. The curved vertical red lines denote the area around the tandem repeated region in the control region, which was omitted from analyses and therefore cut from this plot.

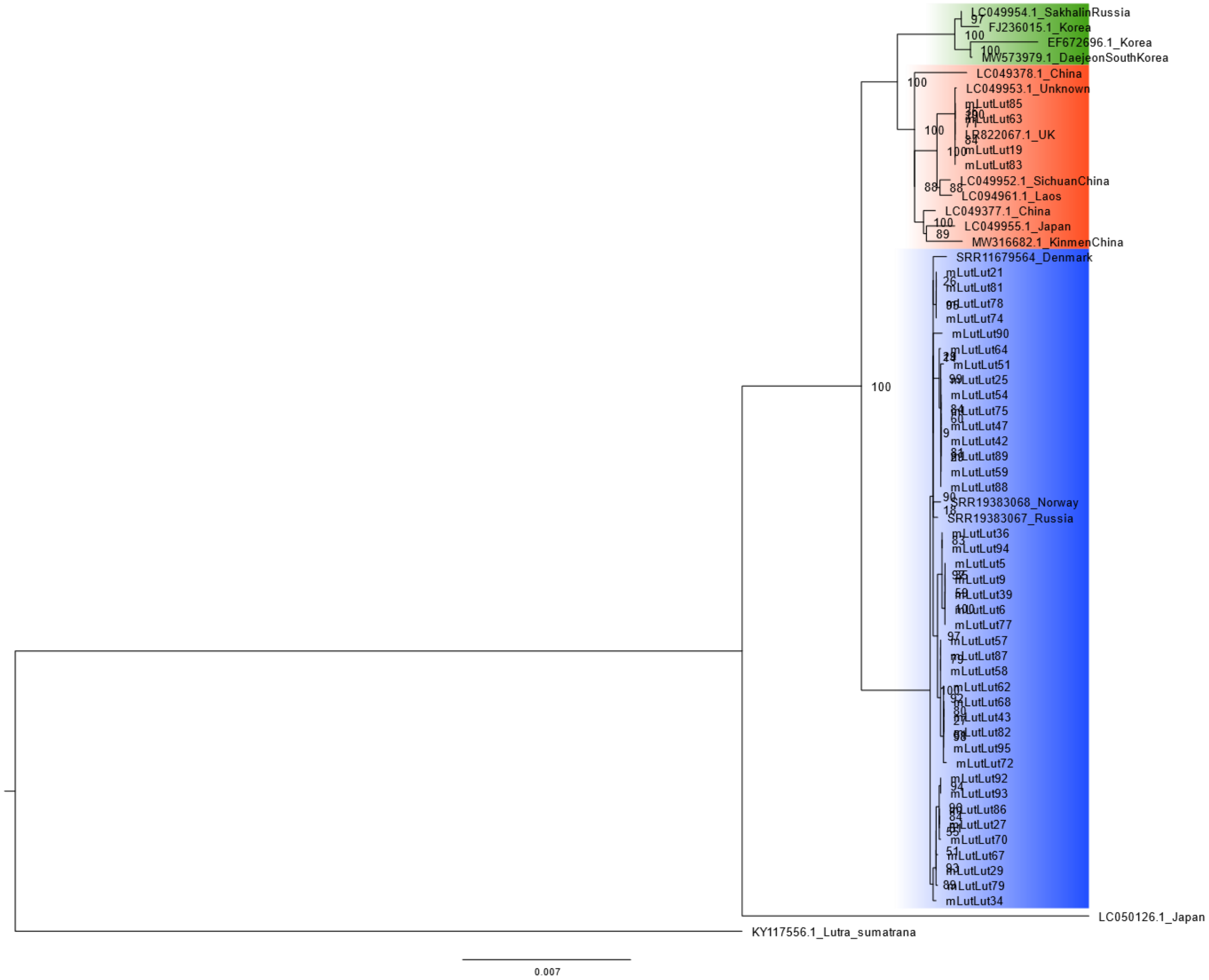

Figure 16. Maximum likelihood phylogeny from IQ-TREE of whole mitochondrial genomes in Eurasian otters. Phylogeny of 16,392 bp whole mitochondrial sequence (with the repeat region removed) generated in this study (n=44), assembled from SRA (n=3), and downloaded from GenBank (n=13), rooted with a Hairy-nosed otter (*Lutra sumatrana*), totalling 61 sequences. Nodes labelled with bootstrap support values, and tree shading indicates lineage 1 (red), 2 (green) and 3 (blue)

12 Runs of Homozygosity methods

Due to the ability of a single incorrectly called variant to break a ROH, variants were filtered separately for these analyses. bcftools was used to split the whole population vcf (autosomal, biallelic SNPs only) into single sample files, allowing each to be filtered to only include SNPs more than half and less than double the average coverage for that sample, as calculated in samtools v1.10 (method in SM2.2, and depths in SM1) (Danecek et al. 2021). SNPs not meeting these criteria were replaced as missing data. Only SNPs with a quality score of over 30 across all samples were included, resulting in a total of 4,933,556 SNPs used in these ROH analyses. SNPs were then converted to the Oxford GEN format per population using bcftools.

For ROH identification, we used RzooRoH v0.3.1 (Druet and Gautier 2017; Bertrand et al. 2019), an R package which uses a hidden Markov model (HMM) to model homozygous-by-descent (HBD) segments of the genome. The length of these segments depends on the number of generations since the common ancestor of the haplotypes, with longer segments indicating recent inbreeding, and the proportion of the genome in HBD segments, the inbreeding coefficient, indicating the extent of inbreeding. We set RzooRoH to model HBD segments in 9 HBD classes (K), with k rates (R_k) of 8, 16, 32, 64, 128, 256, 512, 1024, and 2048, to represent 4, 8, 16, 32, 64, 128, 256, 512, and 1024 generations ago, alongside identifying non-HBD segments. To select this model, we compared models incorporating an error term of 0.01 (increasing the probability of observing a heterozygous genotype in a HBD segment from the default of 0.001) and modelled 6 HBD classes with R_k from 4 to 5120, to 15 HBD classes with R_k from 2 to 5120, to assess the impact of increasing density and range of classes. Bayesian Information Criterion values were used to identify the most suitable model. RzooROH is robust to low-coverage and low-density SNP datasets, and therefore using high coverage, whole genome data, we found filtering had little impact on the results, suggesting we observed a consistent, biologically relevant signal and not the effects of data filtering (Druet and Gautier 2017).

13 References

Altschul, S. F., Gish, W., Miller, W., Myers, E. W. and Lipman, D. J. 1990. Basic local alignment search tool. *Journal of molecular biology* 215(3), pp. 403-410.

Arrendal, J., Vila, C. and Björklund, M. 2007. Reliability of noninvasive genetic census of otters compared to field censuses. *Conservation Genetics* 8(5), pp. 1097-1107.

Beichman, A. C. et al. 2019. Aquatic Adaptation and Depleted Diversity: A Deep Dive into the Genomes of the Sea Otter and Giant Otter. *Molecular Biology and Evolution* 36(12), pp. 2631-2655. doi: 10.1093/molbev/msz101

Bertrand, A. R., Kadri, N. K., Flori, L., Gautier, M. and Druet, T. 2019. RZooRoH: an R package to characterize individual genomic autozygosity and identify homozygous-by-descent segments. *Methods in Ecology and Evolution* 10(6), pp. 860-866.

Bolger, A. M., Lohse, M. and Usadel, B. 2014. Trimmomatic: a flexible trimmer for Illumina sequence data. *Bioinformatics* 30(15), pp. 2114-2120.

Cassens, I., Tiedemann, R., Suchentrunk, F. and Hartl, G. B. 2000. Mitochondrial DNA variation in the European otter (Lutra lutra) and the use of spatial autocorrelation analysis in conservation. *Journal of Heredity* 91(1), pp. 31-35. doi: 10.1093/jhered/91.1.31

Chen, S., Zhou, Y., Chen, Y. and Gu, J. 2018. fastp: an ultra-fast all-in-one FASTQ preprocessor. *Bioinformatics* 34(17), pp. i884-i890.

Chevreux, B., Wetter, T. and Suhai, S. eds. 1999. *Genome sequence assembly using trace signals and additional sequence information*. *German conference on bioinformatics.* Citeseer.

Clement, M., Snell, Q., Walker, P., Posada, D. and Crandall, K. eds. 2002. *TCS: estimating gene genealogies*. *Parallel and Distributed Processing Symposium, International.* IEEE Computer Society.

Crawford, A. and Scholey, G. 2010. *Fifth Otter Survey of England 2009-2010.* Environment Agency Technical Report:

Danecek, P. et al. 2021. Twelve years of SAMtools and BCFtools. *Gigascience* 10(2), doi: 10.1093/gigascience/giab008

de Ferran, V. et al. 2022. Phylogenomics of the world's otters. *Current Biology*,

Dierckxsens, N., Mardulyn, P. and Smits, G. 2017. NOVOPlasty: de novo assembly of organelle genomes from whole genome data. *Nucleic acids research* 45(4), pp. e18-e18.

Druet, T. and Gautier, M. 2017. A model-based approach to characterize individual inbreeding at both global and local genomic scales. *Molecular Ecology* 26(20), pp. 5820-5841.

Edgar, R. C. 2004. MUSCLE: a multiple sequence alignment method with reduced time and space complexity. *Bmc Bioinformatics* 5(1), pp. 1-19.

Finnegan, L. A. and Neill, L. O. 2010. Mitochondrial DNA diversity of the Irish otter, Lutra lutra, population. *Conservation Genetics* 11(4), pp. 1573-1577. doi: 10.1007/s10592-009-9955-4

Formenti, G. et al. 2021. Complete vertebrate mitogenomes reveal widespread repeats and gene duplications. *Genome Biology* 22(1), pp. 1-22.

Hahn, C., Bachmann, L. and Chevreux, B. 2013. Reconstructing mitochondrial genomes directly from genomic next-generation sequencing reads—a baiting and iterative mapping approach. *Nucleic Acids Research* 41(13), pp. e129-e129.

Hauer, S., Ansorge, H. and Zinke, O. 2002. Reproductive performance of otters Lutra lutra (Linnaeus, 1758) in Eastern Germany: low reproduction in a long-term strategy. *Biological Journal of the Linnean Society* 77(3), pp. 329-340.

Heggberget, T. M. 1988. Reproduction in the female European otter in central and northern Norway. *Journal of Mammalogy* 69(1), pp. 164-167.

Hoang, D. T., Chernomor, O., Von Haeseler, A., Minh, B. Q. and Vinh, L. S. 2018. UFBoot2: improving the ultrafast bootstrap approximation. *Molecular Biology and Evolution* 35(2), pp. 518-522.

Hobbs, G. I., Chadwick, E. A., Slater, F. M. and Bruford, M. W. 2006. Landscape Genetics Applied to a Recovering Otter (*Lutra lutra*) Population in the UK: Preliminary Results and Potential Methodologies. *Hystrix, the Italian Journal of Mammalogy* 17(1), pp. 47-63.

Kalyaanamoorthy, S., Minh, B. Q., Wong, T. K., Von Haeseler, A. and Jermiin, L. S. 2017. ModelFinder: fast model selection for accurate phylogenetic estimates. *Nature methods* 14(6), pp. 587-589.

Kean, E. F. and Chadwick, E. A. 2021. *Sixth Otter Survey of Wales 2015-18*. NRW Report No: 519, NRW:

Kimura, M. 1981. Estimation of evolutionary distances between homologous nucleotide sequences. *Proceedings of the National Academy of Sciences* 78(1), pp. 454-458.

Lawson, D. J., Hellenthal, G., Myers, S. and Falush, D. 2012. Inference of population structure using dense haplotype data. *Plos Genetics* 8(1), p. e1002453.

Leigh, J. W. and Bryant, D. 2015. POPART: full-feature software for haplotype network construction. *Methods in Ecology and Evolution* 6(9), pp. 1110-1116.

Li, H. and Durbin, R. 2009. Fast and accurate short read alignment with Burrows–Wheeler transform. *Bioinformatics* 25(14), pp. 1754-1760.

Li, H. and Durbin, R. 2011. Inference of human population history from individual whole-genome sequences. *Nature* 475(7357), pp. 493-U484. doi: 10.1038/nature10231

Mead, D. et al. 2020. The genome sequence of the Eurasian river otter, Lutra lutra Linnaeus 1758. *Wellcome Open Research* 5, doi: 10.12688/wellcomeopenres.15722.1

Nguyen, L.-T., Schmidt, H. A., Von Haeseler, A. and Minh, B. Q. 2015. IQ-TREE: a fast and effective stochastic algorithm for estimating maximum-likelihood phylogenies. *Molecular Biology and Evolution* 32(1), pp. 268-274.

Paradis, E. 2010. pegas: an R package for population genetics with an integrated–modular approach. *Bioinformatics* 26(3), pp. 419-420.

Paradis, E. and Schliep, K. 2019. ape 5.0: an environment for modern phylogenetics and evolutionary analyses in R. *Bioinformatics* 35(3), pp. 526-528.

Pertoldi, C., Hansen, M. M., Loeschcke, V., Madsen, A. B., Jacobsen, L. and Baagoe, H. 2001. Genetic consequences of population decline in the European otter (Lutra lutra): an assessment of microsatellite DNA variation in Danish otters from 1883 to 1993. *Proceedings of the Royal Society B-Biological Sciences* 268(1478), pp. 1775-1781. doi: 10.1098/rspb.2001.1762

Poplin, R. et al. 2018. A universal SNP and small-indel variant caller using deep neural networks. *Nature Biotechnology* 36(10), pp. 983-+. doi: 10.1038/nbt.4235

Pountney, A. 2008. Analysis of the Population Genetics and Polybrominated Diphenyl Ether (PBDE) Burdens of Otters in England and Wales: With Case Studies of Populations in South West England.

Santiago, E., Novo, I., Pardinas, A. F., Saura, M., Wang, J. L. and Caballero, A. 2020. Recent Demographic History Inferred by High-Resolution Analysis of Linkage Disequilibrium. *Molecular Biology and Evolution* 37(12), pp. 3642-3653. doi: 10.1093/molbev/msaa169

Sherrard-Smith, E. and Chadwick, E. A. 2010. Age Structure of the Otter (L*utra lutra*) Population in England and Wales, and Problems with Cementum Ageing. *IU CN Otter Spec. Group Bull.* 27(1), pp. 42-49.

Stanton, D. W. G., Hobbs, G. I., Chadwick, E. A., Slater, F. M. and Bruford, M. W. 2009. Mitochondrial genetic diversity and structure of the European otter (Lutra lutra) in Britain. *Conservation Genetics* 10(3), pp. 733-737. doi: 10.1007/s10592-008-9633-y

Stanton, D. W. G. et al. 2014. Contrasting genetic structure of the Eurasian otter (Lutra lutra) across a latitudinal divide. *Journal of Mammalogy* 95(4), pp. 814-823. doi: 10.1644/13-mamm-a-201

Strachan, R., Birks, J. D. S., Chanin, P. R. F. and J., J. D. 1990. Otter Survey of England 1984-1986. *JNCC*,

Tison, J. L., Blennow, V., Palkopoulou, E., Gustafsson, P., Roos, A. and Dalen, L. 2015. Population structure and recent temporal changes in genetic variation in Eurasian otters from Sweden. *Conservation Genetics* 16(2), pp. 371-384. doi: 10.1007/s10592-014-0664-2

Wong, A. K. et al. 2010. A comprehensive linkage map of the dog genome. *Genetics* 184(2), pp. 595-605.

Yun, T., Li, H., Chang, P. C., Lin, M. F., Carroll, A. and McLean, C. Y. 2020. Accurate, scalable cohort variant calls using DeepVariant and GLnexus. *Bioinformatics* 36(24), pp. 5582-5589. doi: 10.1093/bioinformatics/btaa1081
